# Qianjinweijingtang Decoction and Its Granule Inhibit Respiratory Syncytial Virus Infection and the Associated Inflammatory Response

**DOI:** 10.1002/cbdv.71731

**Published:** 2026-09-20

**Authors:** Chu‐Yi Liao, Mei‐Lan Yang, Ze‐Ping Chen, Bin Chen, Yu‐Jun Tang, Zhen Wang, Hui‐Nan Zhao, Su‐Hua Deng, Jin‐Hai Ye, Zhong Liu, Li‐Feng Chen, Man‐Mei Li

**Affiliations:** ^1^ State Key Laboratory of Bioactive Molecules and Druggability Assessment & College of Pharmacy Jinan University Guangzhou China; ^2^ Guangdong Provincial Key Laboratory of Bioengineering Medicine National Engineering Research Center of Genetic Medicine Institute of Biomedicine College of Life Science and Technology Jinan University Guangzhou China; ^3^ Huizhou No.2 Maternal and Child Health Hospital Huizhou China; ^4^ Longchuan County Maternal and Child Health Hospital Heyuan China; ^5^ Key Laboratory of Innovative Technology Research on Natural Products and Cosmetics Raw Materials Guangzhou China; ^6^ Department of Biochemistry & School of Basic Medical Sciences Guangzhou University of Chinese Medicine Guangzhou China; ^7^ State Key Laboratory of Mechanism and Quality of Chinese Medicine Macau University of Science and Technology Macau China

**Keywords:** bioactive compounds, HIF‐1α, QJWJT, RSV, UHPLC‐Q‐Orbitrap HRMS

## Abstract

Respiratory syncytial virus infection (RSV) represents a leading cause behind acute lower respiratory infections affecting both children and older adults. Qianjinweijingtang (QJWJT), a classical Chinese herbal formula widely used for respiratory diseases, was investigated in this study to compare the anti‐RSV efficacy and chemical profiles of its traditional decoction and granule formulation, and to elucidate the underlying mechanisms. Both formulations significantly inhibited RSV infection and its induced inflammatory responses in vitro and in vivo. Further mechanistic analysis revealed that QJWJT acted at an early step of the RSV replication cycle, most likely by interfering with viral entry. UHPLC‐Q‐Orbitrap HRMS analysis revealed that QJWJT was mainly composed of fatty acids, flavonoids, and phenolic acids, with isoliquiritigenin, quercetin, and gallic acid identified as key bioactive constituents. Moreover, all three compounds bound favorably to both the respiratory syncytial virus fusion (RSV‐F) and glycoprotein (RSV‐G). Network pharmacology combined with western blotting analysis indicated that QJWJT could inhibit RSV infection by downregulating Hypoxia‐Inducible Factor 1‐alpha (HIF‐1α) expression. In conclusion, this study suggests that the granule formulation has the potential to serve as another option for the traditional decoction, providing a reference for the modernization of Chinese herbal formulas.

## Graphical abstract

The classical herbal formula Qianjinweijingtang in both decoction and granule forms, exhibits anti‐RSV activity. Isoliquiritigenin, quercetin, and gallic acid are identified as key active compounds. The mechanism involves interfering with viral entry and downregulating HIF‐1α and inflammatory responses, positioning the granule formulation as a promising modern alternative to the traditional decoction.

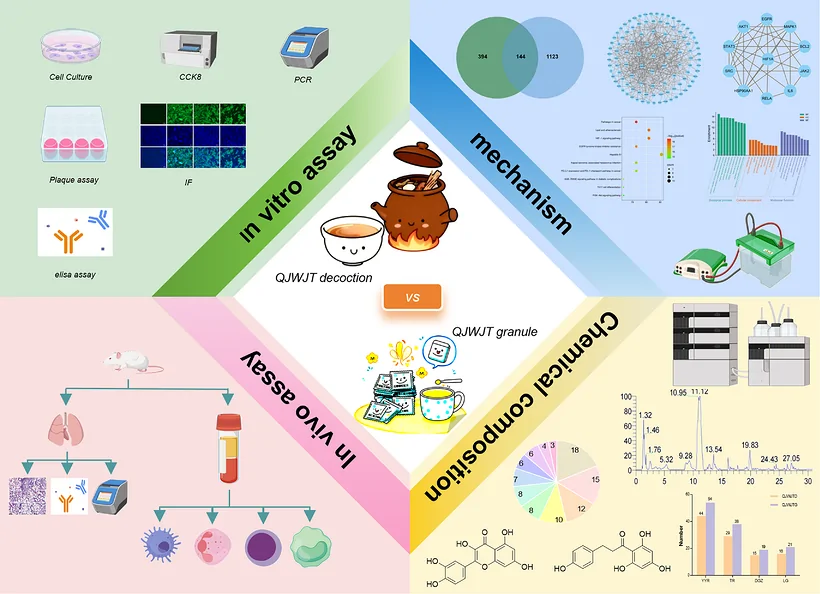

## 1 Introduction

A member of the Pneumoviridae family, Respiratory syncytial virus infection (RSV) is a pleomorphic, enveloped RNA virus that ranks as a primary pathogen responsible for bronchiolitis and pneumonia. These lower respiratory tract infections occur most frequently in infants, older adults, and immunocompromised populations [1, 2, 3]. RSV‐linked acute lower respiratory infections account for roughly 33.0 million episodes, 3.6 million hospital admissions, and 26,300 inpatient fatalities among children younger than five [4]. Despite this substantial burden, treatments against RSV are scarce. Current interventions — such as supportive care, palivizumab, ribavirin, and the recently approved vaccines (Arexvy, Abrysvo, and mRNA‐based candidates) — are hampered by narrow indications, suboptimal efficacy, safety concerns, and prohibitive costs. These limitations underscore an urgent need to develop novel, safe, and more accessible anti‐RSV strategies [5, 6, 7, 8].

RSV infection is critically shaped by the immune system [7]. Upon viral invasion, immune cells rapidly produce and secrete pro‐inflammatory cytokines and chemokines to contain viral replication and eliminate the pathogen [9]. However, an exaggerated or dysregulated immune response can paradoxically enhance viral replication and amplify inflammatory cascades, leading to airway obstruction, mucus hypersecretion, and mucosal edema in the lower respiratory tract. Progressive aggravation of these pathological changes may culminate in severe pneumonia and even fatal outcomes [10, 11]. Accordingly, suppressing excessive inflammatory cytokine production has emerged as a promising therapeutic strategy for RSV infection [12, 13].

Traditional Chinese Medicine (TCM) has long been used to treat respiratory diseases. Qianjinweijingtang (QJWJT), a classic formula documented in *Gujin Luyan*, consists of *Phragmites australis* (Cav.) Steud., *Benincasa hispida* Cogn. seed, *Prunus persica* (L.) Batsch. seed, and *Coix lacryma‐jobi var. ma‐yuen* (Rom.Caill.) Stapf. seed (plant names validated with World Flora Online, http://www.worldfloraonline.org) [14]. Pharmacologically, QJWJT exhibits a broad spectrum of activities, including immunomodulation, anti‐inflammation, pulmonary protection, and tissue repair, and has been clinically applied in conditions such as COVID‐19, mycoplasma and bacterial pneumonia, and acute/chronic bronchitis [15, 16, 17, 18]. Despite increasing evidence supporting its therapeutic potential in respiratory disorders, the precise molecular mechanisms underlying its effects remain largely undefined. Moreover, its antiviral activity against RSV has yet to be systematically investigated.

TCM formula granules represent a modernized dosage form developed in accordance with TCM theory. They are prepared via extraction, concentration, drying, and granulation, offering advantages such as ease of administration, portability, precise dosing and enhanced stability. Despite these benefits, a unified national quality standard for QJWJT granule has yet to be established, and whether its therapeutic efficacy differs from that of the traditional decoction remains unclear.

This study aimed to comparatively evaluate the anti‐RSV efficacy of QJWJT decoction (QJWJTD) and granule (QJWJTG) through integrated in vitro and in vivo experiments, characterize their chemical constituents to identify the pharmacodynamic material basis using UHPLC‐Q‐Orbitrap HRMS and elucidate the underlying mechanisms via network pharmacology combined with experimental validation. These findings support QJWJT granule as a modern, mechanism‐based alternative to the traditional decoction.

## 2 Results and Discussion

### 2.1 Results

#### 2.1.1 QJWJT Inhibits RSV Infection In Vitro

**FIGURE 1:**
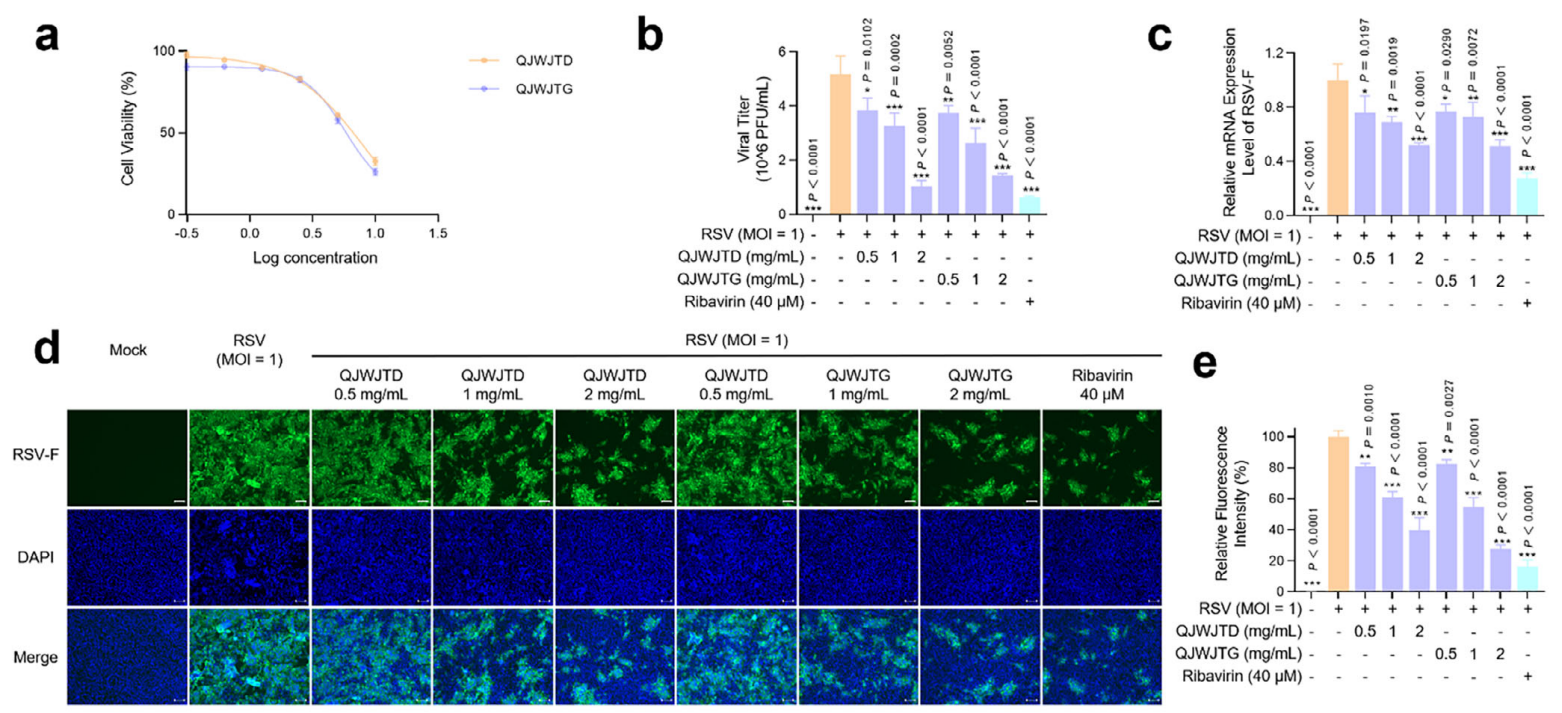
QJWJT inhibits RSV infection in vitro. Hep‐2 cells were mock‐infected or infected with RSV (MOI = 1) for 48 h. (a) Cytotoxicity of QJWJT against Hep‐2 cells was measured using CCK‐8 assay. (b) Plaque assay for anti‐RSV activity of QJWJT. (c) The effect of QJWJT on the mRNA expression of RSV‐F was measured by RT‐qPCR. (d) The effect of QJWJT on the expression of RSV‐F was measured by Immunofluorescence assay. (e) Quantitative analysis by Immunofluorescence assay. Data are presented as mean ± SD (n = 3), statistical analysis using one‐way ANOVA. Compared with the RSV group, *P < 0.05, **P < 0.01, ***P < 0.001.

The cytotoxic effects of QJWJTD and QJWJTG were assessed in Hep‐2 cells using the cell counting kit‐8 (CCK‐8) assay. As shown in Figure 1a, the cytotoxic concentration 50% (CC_50_) of QJWJTD and QJWJTG were 6.81 mg/mL and 5.75 mg/mL, respectively. Based on these noncytotoxic concentrations, subsequent antiviral assays were conducted at 2, 1, and 0.5 mg/mL. Ribavirin, a broad‐spectrum antiviral agent with established activity against RSV, was used as the positive control. The results showed that viral titers were significantly reduced by both QJWJTD and QJWJTG in a concentration‐dependent manner (Figure 1b), indicating effective inhibition of RSV replication. Consistently, reverse transcription quantitative polymerase chain reaction (RT‐qPCR) analysis and immunofluorescence staining demonstrated that both formulations remarkably suppressed the mRNA and protein expression of respiratory syncytial virus fusion protein (RSV‐F) (*p* < 0.001) (Figure 1c–e). Collectively, these results demonstrate that both QJWJTD and QJWJTG effectively inhibit RSV replication in vitro.

The RSV replication cycle consists of viral entry, genome replication, assembly, and budding. To determine the step targeted by QJWJT, we performed a time‐of‐addition assay. As shown in Figure S1, green fluorescence (indicating the RSV‐F protein) was abundant in the untreated virus‐infected control. Both QJWJTD and QJWJTG significantly inhibited RSV infection when added during viral inoculation (0–2 h post‐infection). In contrast, addition of the compounds later than 2 h post‐infection resulted in fluorescence intensities that did not differ significantly from those of the virus control group. These results demonstrate that QJWJTD and QJWJTG act at an early step of the RSV replication cycle, most likely by interfering with viral entry.

#### 2.1.2 QJWJT Inhibits RSV‐Induced Inflammation In Vitro

**FIGURE 2:**
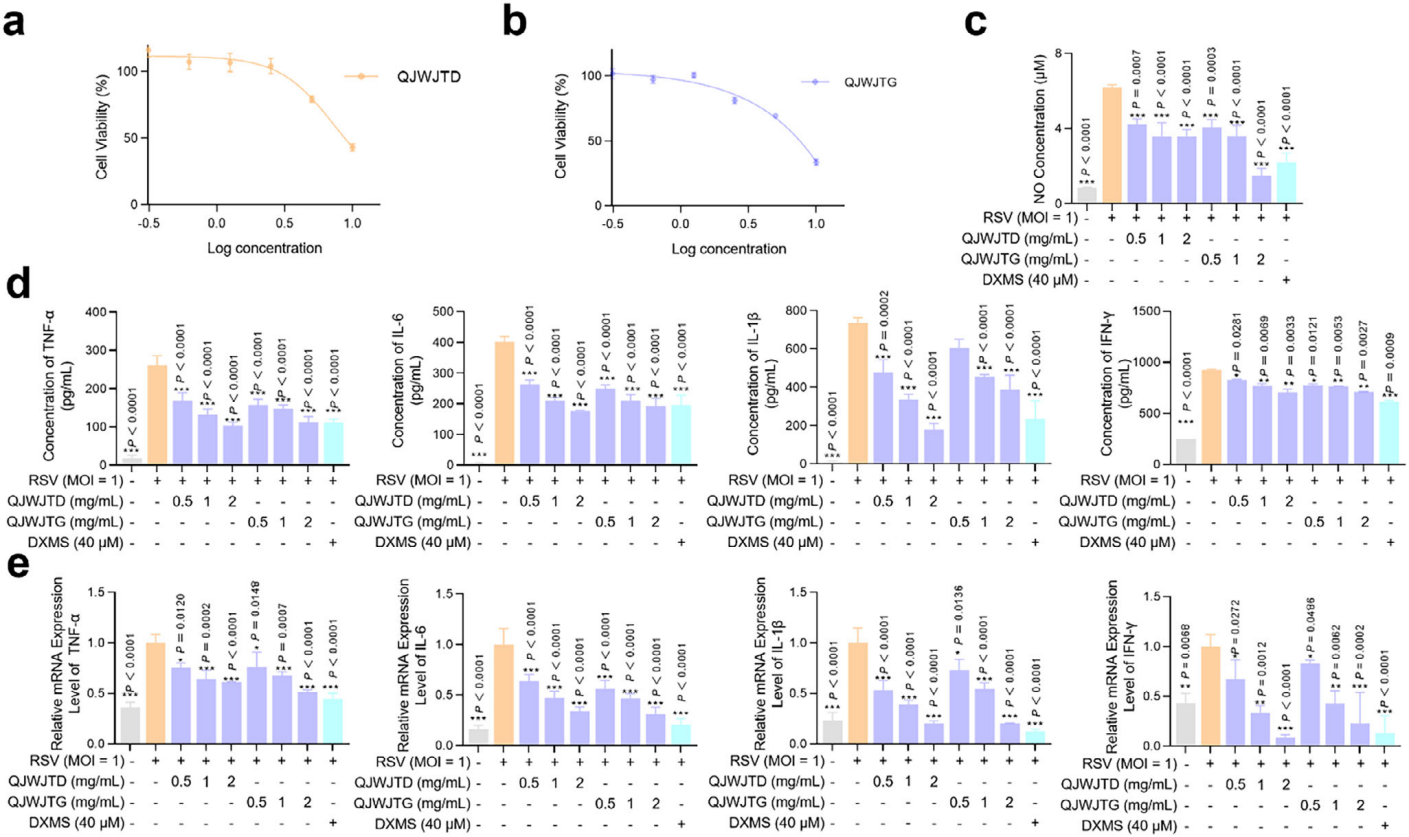
QJWJT inhibits RSV‐induced inflammation in vitro. RAW264.7 cells were mock‐infected or infected with RSV (MOI = 1) for 24 h. (a‐b) Cytotoxicity of QJWJT against RAW264.7 cells was measured using CCK‐8 assay. (c) The effect of QJWJT on RSV‐induced NO production was detected using NO production assay. (d) The effect of QJWJT on the protein expression of TNF‐α, IL‐6, IL‐1β, and IFN‐γ was measured by ELISA, respectively. (e) The effect of QJWJT on the mRNA expression of TNF‐α, IL‐6, IL‐1β and IFN‐γ was measured by RT‐qPCR. Data are presented as mean ± SD (n = 3), statistical analysis using one‐way ANOVA. Compared with the RSV group, *P < 0.05, **P < 0.01, ***P < 0.001.

The cytotoxicity of QJWJTD and QJWJTG against RAW264.7 cells was detected using the CCK‐8 assay. Their respective CC_50_ values were 8.65 mg/mL and 7.52 mg/mL (Figure 2a,b). Next, the anti‐inflammation activity of QJWJTD and QJWJTG against RSV‐induced inflammation was further evaluated by RT‐qPCR and enzyme‐Linked immunosorbent assay (ELISA). The Dexamethasone (DXMS), a potent anti‐inflammatory corticosteroid, served as the positive control for the inhibition of RSV‐induced inflammatory responses. Compared with the RSV‐infected group, both QJWJTD and QJWJTG significantly reduced RSV‐induced production of nitric oxide (NO), tumor necrosis factor‐alpha (TNF‐α), interleukin‐6 (IL‐6), interleukin‐1 beta (IL‐1β), and interferon‐gamma (IFN‐γ) in a dose‐dependent manner (Figure 2c–e; *p* < 0.001). The inhibitory effects of the 2 mg/mL dose group were comparable to those of the DXMS positive control. These results suggest that QJWJT inhibits RSV‐induced inflammation in vitro.

#### 2.1.3 QJWJT Inhibits RSV Infection and Associated Inflammation In Vivo

**FIGURE 3:**
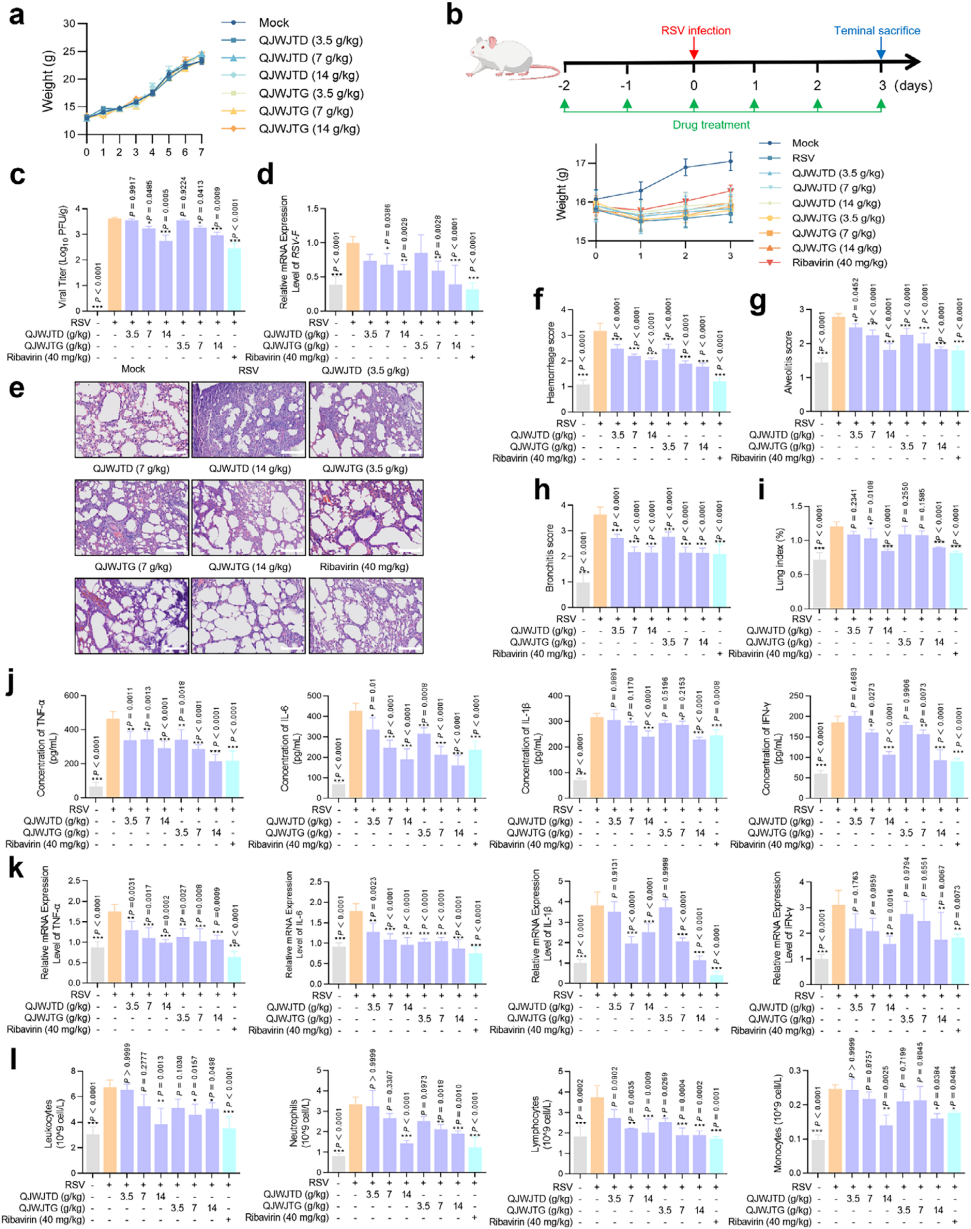
QJWJT inhibits RSV infection and associated inflammation in vivo. (a) Mice were intragastrically administered with normal saline (Mock), QJWJTD, or QJWJTG once daily for 7 consecutive days, and the body weight was recorded daily. (b) Experimental scheme and body weight line chart. BALB/c mice were mock‐infected or infected with RSV. QJWJT or ribavirin was administered intragastrically daily from day ‐2 to day 2 post‐infection. On day 3 post‐infection, lung tissues and plasma were collected for analysis. The mouse element in this figure was created using BioGDP.com (https://BioGDP.com). (c) Viral titers in lung tissues were determined by plaque assay. (d) The effect of QJWJT on the mRNA expression of RSV‐F was measured by RT‐qPCR. (e‐h) Representative H&E staining images and histopathological scores of lung lesions in mice. Scale bar: 200 µm. (i) The lung indices represent the ratio of lung weight to body weight. (j) The effect of QJWJT on the protein expression of TNF‐α, IL‐6, IL‐1β, and IFN‐γ was measured by ELISA. (k) The effect of QJWJT on the mRNA expression of TNF‐α, IL‐6, IL‐1β and IFN‐γ was measured by RT‐qPCR. (l) The number of monocytes, lymphocytes, neutrophils and leukocytes in blood. Data are presented as mean ± SD (n = 6 mice), statistical analysis using one‐way ANOVA. Compared with the RSV group, *P < 0.05, **P < 0.01, ***P < 0.001.

To preliminarily assess their potential toxicity in vivo, QJWJTD and QJWJTG were administered by gavage to healthy mice for 7 consecutive days, with daily monitoring of body weight. Compared with the normal saline control group, neither QJWJTD nor QJWJTG caused significant body weight loss over the 7‐day period, indicating that the tested doses did not induce acute toxicity or adversely affect body weight (Figure 3a). Next, a BALB/c mouse model of RSV infection was established to evaluate the in vivo antiviral efficacy of QJWJTD and QJWJTG, with ribavirin serving as the positive control. RSV infection caused marked body weight loss; however, treatment with QJWJTD or QJWJTG (3.5, 7, and 14 g/kg) did not significantly alleviate this loss (Figure 3b). Viral titers and RSV‐F mRNA levels in lung tissues were markedly elevated following RSV challenge. In contrast, both QJWJTD and QJWJTG reduced these parameters in a dose‐dependent manner, with significant effects observed at 7 and 14 g/kg (Figure 3c,d). These results suggest that QJWJT inhibits RSV infection in vivo.

The lung index (lung wet weight/body weight) is a commonly used indicator for evaluating pulmonary inflammation. RSV infection triggers immune responses via viral replication, leading to inflammatory cell infiltration and pulmonary edema. These pathological changes increase lung wet weight and consequently elevate the lung index. As shown in Figure 3i, the lung index in RSV‐infected mice was significantly higher than that in the control group, indicating that the model successfully induces pneumonia in mice. QJWJTD and QJWJTG treatments decreased the lung index in a dose‐dependent manner, with a significant effect observed at 14 g/kg. Histopathological examination by hematoxylin and eosin (H&E) staining revealed that QJWJTD or QJWJTG treatment alleviated RSV‐induced lung injury with decreased inflammatory infiltration and attenuated alveolar wall thickening (Figure 3e–h). Consistently, the results of RT‐qPCR and ELISA showed that the mRNA and protein levels of TNF‐α, IL‐6, IL‐1β, and IFN‐γ in lung tissues were significantly upregulated in the RSV‐infected group compared with the normal control group. Treatment with QJWJTD or QJWJTG (3.5–14 g/kg) led to a dose‐dependent decrease in the expression of these inflammatory cytokines (Figure 3j,k), suggesting that QJWJT exerts anti‐inflammatory activity against RSV‐induced inflammation. Peripheral blood analysis revealed that high doses of QJWJTD or QJWJTG treatment attenuated RSV‐induced elevations in monocytes, lymphocytes, neutrophils, and leukocytes (Figure 3l). Collectively, QJWJT inhibits RSV infection and associated inflammation in vivo.

#### 2.1.4 Chemical Profiling of QJWJT by UHPLC‐Q‐Orbitrap HRMS

**FIGURE 4:**
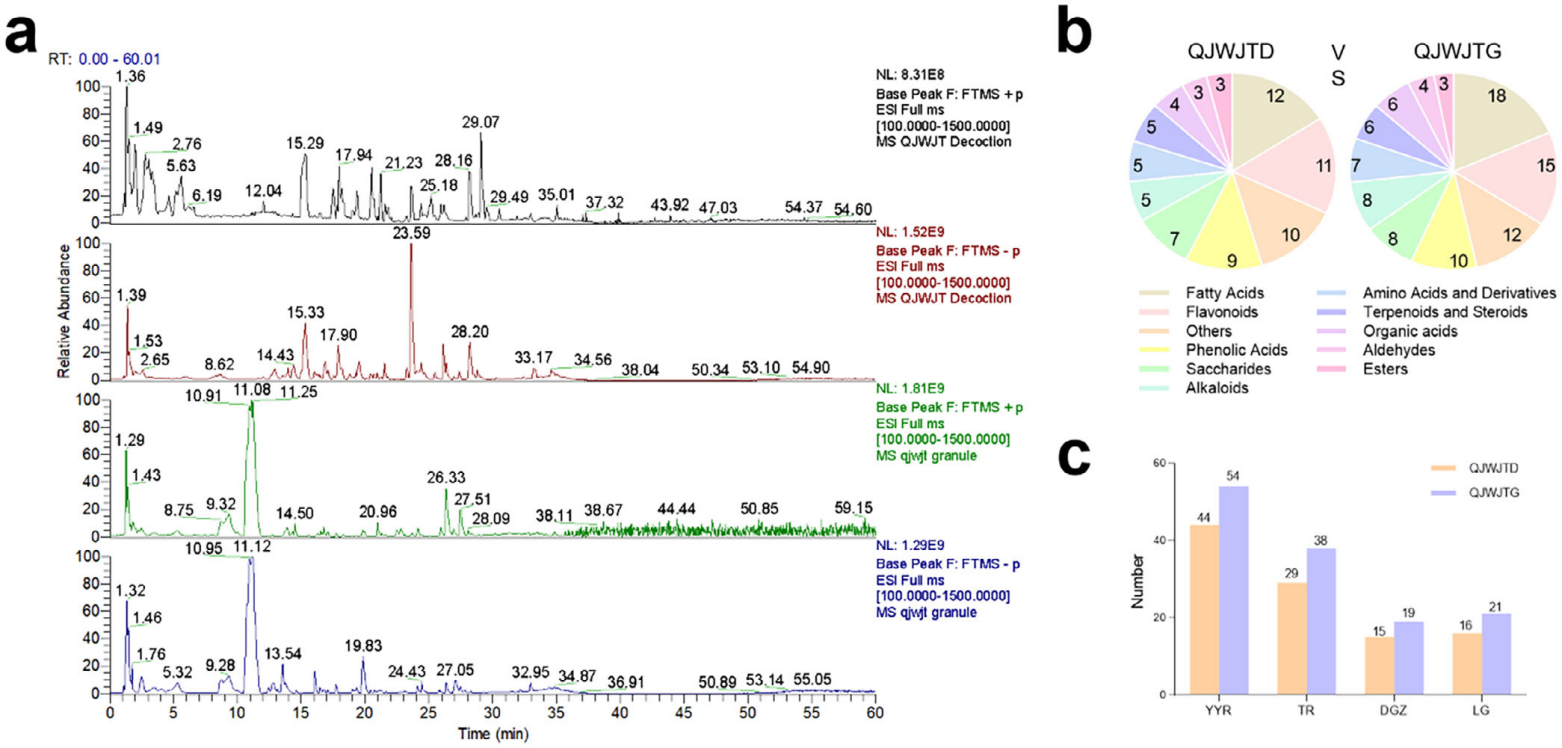
Chemical profiling of QJWJT by UHPLC‐Q‐Orbitrap HRMS. (a) Base peak chromatograms (BPC) of QJWJTD and QJWJTG in positive and negative ion modes. (b) Structural classification of the compounds identified in QJWJTD and QJWJTG. (c) Number of compounds identified from each constituent herb of QJWJT (YYR, Coix Seed; TR, Peach Kernel; DGZ, Waxgourd Seed; LG, Reed Rhizome).

Chemical characterization of QJWJT was performed using UHPLC‐Q‐Orbitrap HRMS (Figure 4a; Tables S1 and S2). A total of seventy‐four compounds were identified in QJWJTD, including nine phenolic acids, twelve fatty acids, and eleven flavonoids. Meanwhile, ninety‐seven compounds were characterized in QJWJTG, including twelve fatty acids, eighteen flavonoids, and fifteen phenolic acids. The two preparations shared fifty‐four common compounds. (Figure 4b). Compound source attribution analysis showed that the numbers of compounds derived from Coix Seed (YYR), Peach Kernel (TR), Waxgourd Seed (DGZ), and Reed Rhizome (LG) were forty‐four, twenty‐nine, fifteen, and sixteen in QJWJTD, and fifty‐four, thirty‐eight, nineteen, and twenty‐one in QJWJTG, respectively (Figure 4c).

#### 2.1.5 Identification of Absorbed Compounds in Mouse Plasma After QJWJT Administration

**FIGURE 5:**
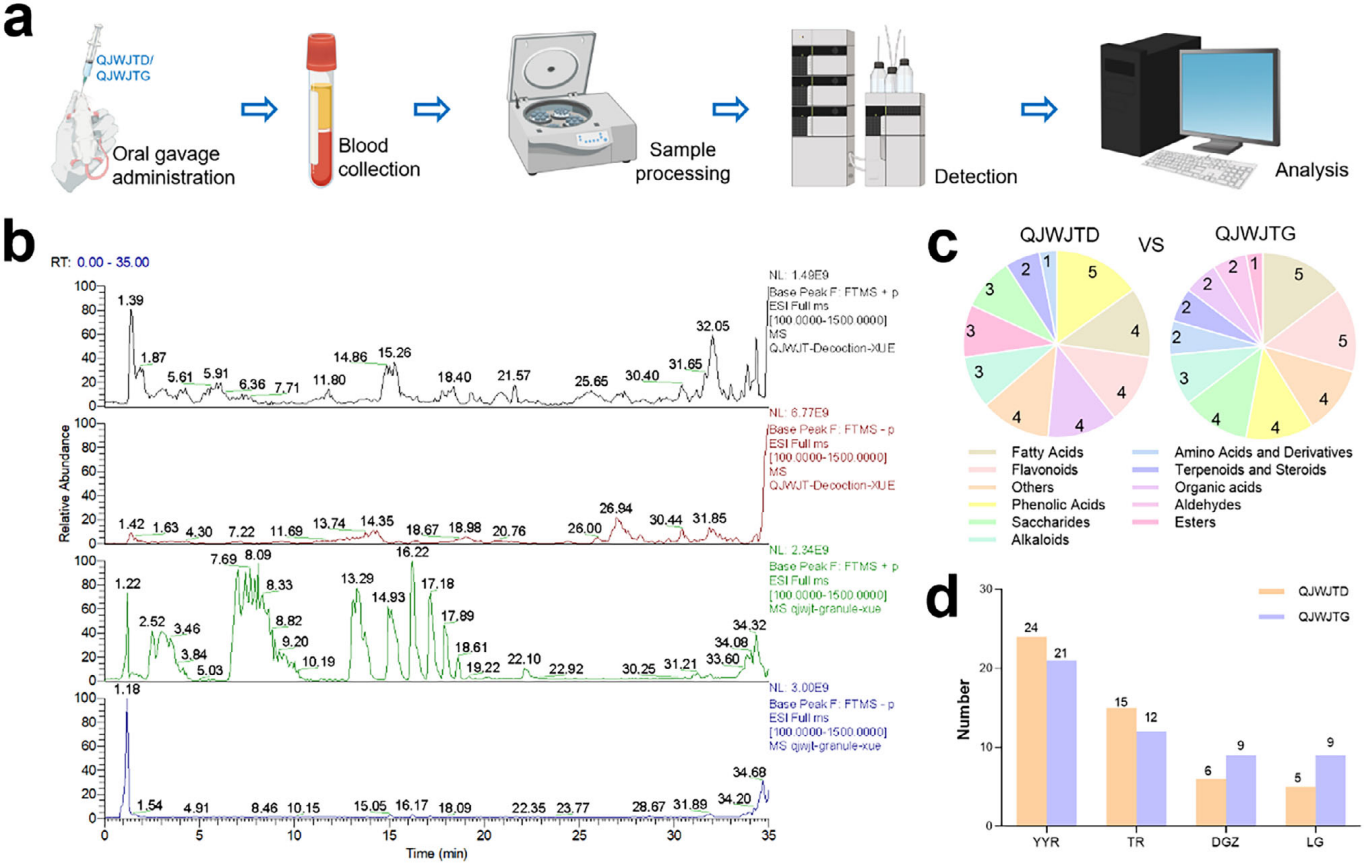
Identification of absorbed compounds in mouse plasma after QJWJT administration. (a) Workflow for the identification of absorbed constituents in mouse plasma. This part was created using BioGDP.com (https://BioGDP.com). (b) Base peak chromatograms (BPC) of mouse plasma following administration of QJWJTD or QJWJTG, in positive and negative ion modes. (c) Structural classification of the absorbed compounds derived from QJWJTD and QJWJTG. (d) Number of absorbed compounds identified from each constituent herb of QJWJT (YYR, Coix Seed; TR, Peach Kernel; DGZ, Waxgourd Seed; LG, Reed Rhizome).

The plasma of mice was analyzed by UHPLC‐Q‐Orbitrap HRMS to identify the blood‐entry active substances of QJWJT (Figure 5a,b; Tables S3 and S4). After administration of QJWJTD and QJWJTG, thirty‐three and thirty‐four compounds were detected in the blood, respectively, of which twenty‐three were common compounds (Figure 5c,d). Further classification showed that QJWJT mainly consists of fatty acids, flavonoids and phenolic acids, suggesting that the anti‐RSV and anti‐inflammatory effects of QJWJT may be associated with these compounds.

#### 2.1.6 Validation of Bioactive Compounds From QJWJT

**FIGURE 6:**
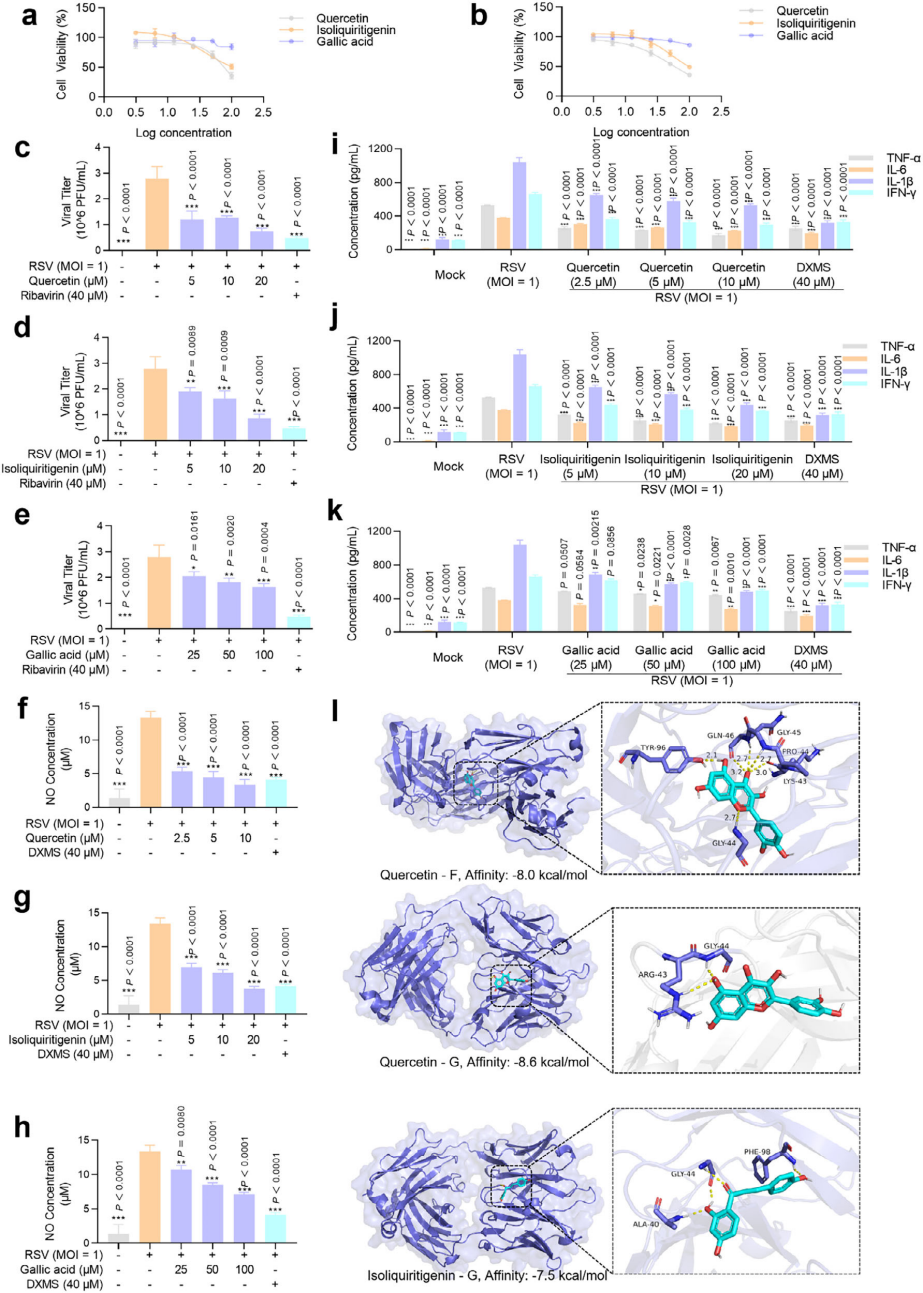
Validation of bioactive compounds from QJWJT. Based on absorbed constituent analysis in mouse plasma and cytopathic effect (CPE) assays, gallic acid, quercetin, and isoliquiritigenin were identified as candidate active components and subjected to further validation. Cytotoxicity of gallic acid, quercetin, and isoliquiritigenin against Hep‐2 cells (a) and RAW264.7 cells (b) was measured using CCK‐8 assay. (c‐e) Viral titers in cells treated with gallic acid, quercetin, or isoliquiritigenin were determined by plaque assay. (f‐h) Effects of the compounds on RSV‐induced NO production were detected by NO assay. (i‐k) Effects of the compounds on TNF‐α, IL‐6, IL‐1β, and IFN‐γ protein expression were measured by ELISA. (l) Detailed views of the binding modes of quercetin with the RSV‐F and G proteins, and isoliquiritigenin with the RSV‐G protein. Data are presented as mean ± SD (n = 3), statistical analysis using one‐way ANOVA. Compared with the RSV group, *P < 0.05, **P < 0.01, ***P < 0.001.

To elucidate the pharmacodynamic basis of QJWJT against RSV infection and its induced inflammation, active components were screened by integrating the identification of absorbed constituents in mouse plasma with a cytopathic effect (CPE) assay. As shown in Figure 6a, the maximum noncytotoxic concentrations of quercetin, isoliquiritigenin, and gallic acid in Hep‐2 cells were 20, 40, and 100 µM, respectively. In RAW264.7 cells, the corresponding concentrations were 10, 20, and 100 µM, respectively (Figure 6b). At noncytotoxic concentrations, these three compounds significantly reduced RSV titers in a dose‐dependent manner (Figure 6c‐e). Furthermore, they markedly suppressed RSV‐induced production of inflammatory factors, including NO, TNF‐α, IL‐6, IL‐1β, and IFN‐γ (Figure 6f‐k). These results indicate that Gallic acid, quercetin, and isoliquiritigenin exhibit inhibitory activity against RSV infection and its induced inflammation in vitro.

Given the critical roles of the RSV‐F and G protein (respiratory syncytial virus glycoprotein) in viral entry, we used molecular docking to evaluate the binding of quercetin, isoliquiritigenin, and gallic acid to these proteins. The docking results revealed that the binding energies of these three compounds with the F protein were −8.0, −6.6, and −6.5 kcal/mol, respectively, and with the G protein were −8.6, −7.5, and −5.9 kcal/mol. All binding energies were below −5.0 kcal/mol, indicating that these three compounds can spontaneously bind to both target proteins. Conformational analysis showed that quercetin occupied the binding pocket of the RSV‐F protein, forming a hydrogen bond network with residues including Tyrosine 96 (TYR‐96) and Glutamine 46 (GLN‐46) and thereby stabilizing the complex. Quercetin exhibited the highest affinity for the RSV‐G protein, with its binding stabilized by hydrogen bonds to Arginine 43 (ARG‐43) and Glycine 44 (GLY‐44) as well as hydrophobic interactions. In contrast, isoliquiritigenin formed only limited hydrogen bonds with the G protein, involving residues Alanine 40 (ALA‐40), Glycine 44 (GLY‐44), and Phenylalanine 98 (PHE‐98), and displayed moderate binding affinity (Figure 6l). Taken together, these results indicate that all three compounds bind favorably to the target proteins and likely represent the key active constituents responsible for the anti‐RSV activity of QJWJT. To further clarify the content differences of the above‐mentioned active components between the decoction and granule forms, we quantitatively analyzed isoliquiritigenin and gallic acid in both formulations. The results showed that the content of isoliquiritigenin was 0.17 mg/g in QJWJTD and 0.11 mg/g in QJWTJG, while that of gallic acid was 4.62 and 4.22 mg/g, respectively (Figure S2). This finding provides a reference for the quality control of QJWJT.

#### 2.1.7 Target Prediction and Verification of QJWJT Against RSV

**FIGURE 7:**
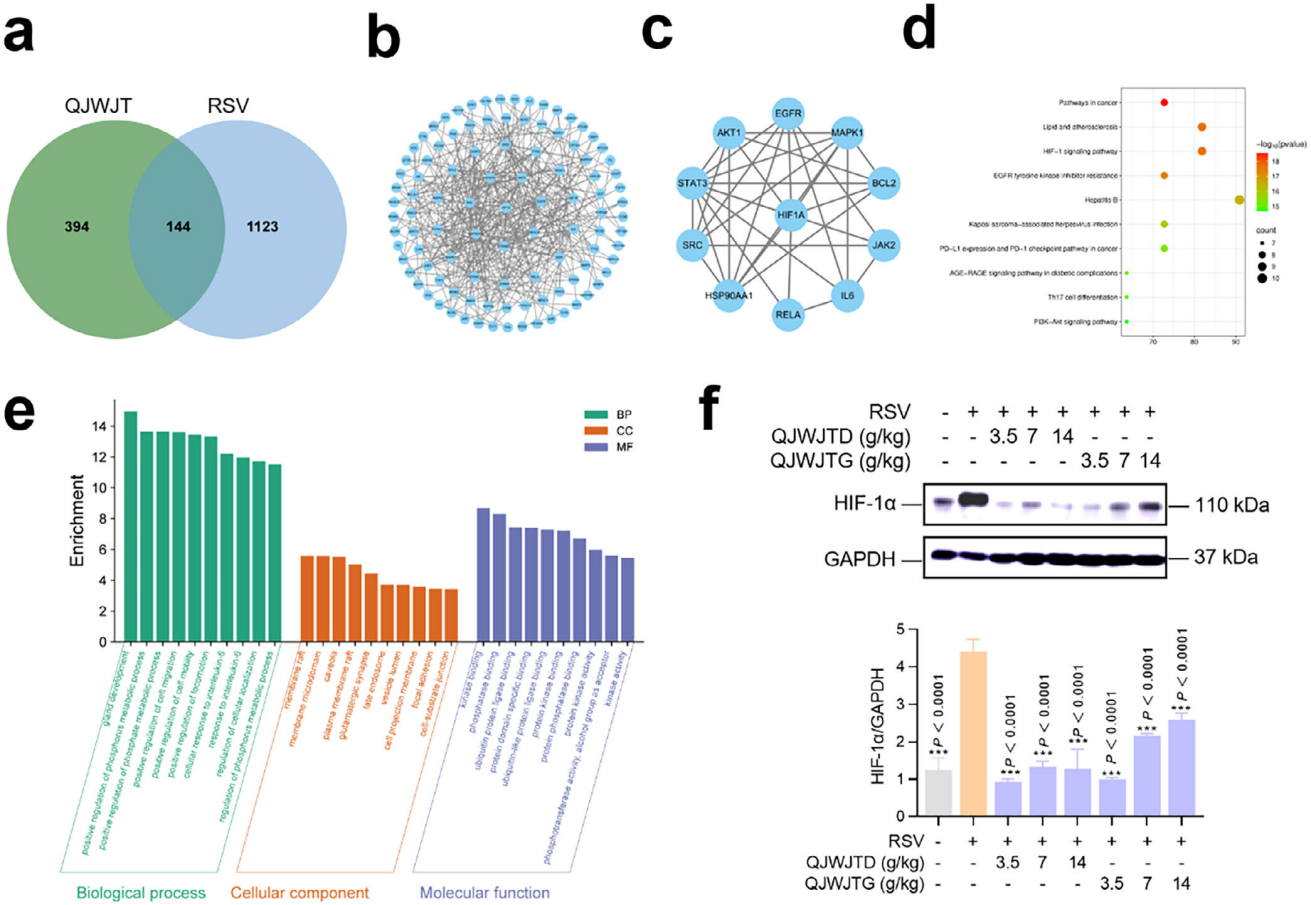
Target prediction and verification of QJWJT against RSV. (a) The venn diagram of QJWJT component protein targets and RSV disease targets. (b) Construction of PPI protein network. (c) Core targets. (d, e) KEGG and GO analysis. (f) Western blotting analysis of the effects of QJWJT on RSV‐induced HIF‐1α expression. Data are presented as mean ± SD (n = 3), statistical analysis using one‐way ANOVA. Data are presented as mean ± SD (n = 3). Compared with the RSV group, *P < 0.05, **P < 0.01, ***P < 0.001.

Network pharmacology was applied to explore QJWJT's anti‐RSV mechanism. A total of 538 protein targets of the blood‐absorbed compounds from QJWJT were identified using the SwissTargetPrediction (probability > 0.1), SEA (Max Tc > 0.5), and STITCH (combined score > 0.7) databases, while 1267 RSV‐related targets were sourced from the GeneCards, OMIM, DrugBank, DisGeNet, and TTD databases. Intersection analysis revealed 144 potential therapeutic targets of QJWJT against RSV (Figure 7a). A protein‐protein interaction (PPI) network was built from these overlapping targets, comprising 126 nodes and 388 edges (confidence > 0.9, Figure 7b). Key targets were screened based on topological parameters, retaining those with Degree, Closeness Centrality (CC), and Betweenness Centrality (BC) values exceeding twice the median. This process yielded 10 hub genes (signal transducer and activator of transcription 3, STAT3; SRC proto‐oncogene, nonreceptor tyrosine kinase, SRC; rela proto‐oncogene, nf‐kb subunit, RELA; mitogen‐activated protein kinase 1, MAPK1; janus kinase 2, JAK2; interleukin 6, IL‐6, heat shock protein 90 alpha family class a member 1, HSP90AA1; hypoxia inducible factor 1 alpha, HIF1α; epidermal growth factor receptor, EGFR; bcl2 apoptosis regulator, BCL2; akt serine/threonine kinase 1, AKT1) with the specific thresholds of Degree > 10, CC > 0.15, and BC > 0.02 (Figure 7c). KEGG pathway enrichment suggested that potential effect of QJWJT against RSV infection may be associated with the HIF‐1 signaling pathway (Figure 7d). GO analyses with Metascape database identified 326 biological processes, 67 molecular functions, and 74 cellular components (Figure 7e). To further verify the above results, western blotting was used to measure HIF‐1α expression. The in vivo results showed that RSV infection induced the upregulation of HIF‐1α expression, and QJWJT treatment effectively inhibited this process (Figure 7f).

## 3 Discussion

TCM has long been applied to both prevent and manage respiratory illnesses. The development of novel TCM formulations, grounded in its theoretical framework, may offer important therapeutic avenues for managing RSV infection and associated viral pneumonia. For instance, Qingfei oral liquid ameliorates RSV‐mediated lung inflammation by facilitating M1/M2 macrophage polarization dependent on fatty acids, with involvement of the Akt signaling axis [19]. Similarly, Sanshibaidu decoction reduces SARS‐CoV‐2 viral load and attenuates lung inflammation in vivo. Furthermore, SJCG has been reported to significantly suppress influenza A virus replication and associated inflammation through modulation of the RIG‐I/NF‐κB/IFN axis [20, 21]. These results underscore the promise of TCM as a viable approach for treating RSV‐induced inflammation. This study aimed to investigate the anti‐RSV effects of QJWJTD and QJWJTG, and compare the differences in efficacy between the two formulations. Our results demonstrated that both QJWJTD and QJWJTG showed significant anti‐RSV replication and anti‐inflammatory activity in vitro and in vivo. Furthermore, QJWJT acted at an early step of the RSV replication cycle, most likely by interfering with viral entry.

UHPLC‐Q‐Orbitrap HRMS analysis revealed that QJWJT is rich in flavonoids, fatty acids, and phenolic acids, which may contribute to its anti‐RSV effects. Previous studies have shown that phloretin significantly reduces viral titers by inhibiting host cell glucose uptake and modulating the Akt/mTOR pathway [22]. Furthermore, chlorogenic acid combats infectious bronchitis virus by modulating innate immunity via the NF‐κB/TLR7/MDA5/ pathways [23]. Additionally, sinapic acid has been reported to inhibit viral replication by targeting the SARS‐CoV‐2 envelope protein [24]. In the present study, we confirmed that isoliquiritigenin, quercetin, and gallic acid inhibited RSV replication and suppressed the RSV‐induced expression of TNF‐α, IL‐6, IL‐1β, and IFN‐γ, suggesting that these compounds are active constituents of QJWJT responsible for its anti‐RSV effects. Furthermore, molecular docking revealed that all three compounds bound favorably to the RSV‐F and G proteins, consistent with the finding that QJWJT acts at viral entry. UHPLC‐Q‐Orbitrap HRMS analysis revealed significant differences in the chemical compositions between QJWJT decoction and granules. Specifically, the relative contents of fatty acids, flavonoids, and phenolic acids in the decoction were 16.22%, 14.87%, and 12.16%, respectively, whereas the corresponding proportions in the granules were 18.56%, 15.46%, and 10.31%, sequentially. Such variations may be attributed to the differences in preparation processes and storage conditions between the two dosage forms [25, 26, 27, 28].

HIF‐1α is a pivotal regulator of immune‐inflammatory responses [29, 30]. Upon nuclear translocation, it binds to hypoxia response elements (HREs) and directly drives the transcription of TNF‐α, IL‐6, and IL‐1β [31, 32, 33]. It further amplifies the expression of these pro‐inflammatory cytokines by upregulating the NLRP3 inflammasome and enhancing NF‐κB transcriptional activity [34]. Consequently, dysregulation of HIF‐1α can disrupt antiviral defense and inflammatory homeostasis [35]. Numerous viruses, including influenza virus, exploit the HIF‐1α pathway to evade immune surveillance or exacerbate inflammatory damage [36]. We previously demonstrated that RSV infection activates HIF‐1α, which in turn triggers the NLRP3/caspase‐1 inflammasome, leading to the release of pro‐inflammatory cytokines (TNF‐α, IL‐6, and IL‐1β) and aggravated pulmonary inflammation and tissue injury [37, 38]. In the present study, we found that QJWJT treatment significantly suppressed RSV‐induced HIF‐1α upregulation and concurrently reduced the secretion of these cytokines. These results suggest that the anti‐inflammatory effect of QJWJT involves blockade of the HIF‐1α/NLRP3/caspase‐1 signaling cascade and that HIF‐1α represents a potential target for QJWJT against RSV. These findings provide novel mechanistic insights into the anti‐inflammatory action of QJWJT, although the precise molecular targets and the complete signaling network remain to be fully elucidated.

## 4 Conclusions

In conclusion, both the traditional decoction and the modern granule formulation of QJWJT effectively inhibit RSV replication and suppress virus‐induced inflammatory responses by regulating HIF‐1α. These findings offer a scientific basis for the clinical application of QJWJT in the management of RSV‐associated respiratory infections and contribute to the modernization of classical TCM formulations.

## 5 Experimental Section

### 5.1 Preparation of QJWJT and Determination of Dosage Regimens

#### 5.1.1 Preparation of QJWJTD

*P. australis* (Cav.) Steud. (LG, Lot: 1088124), *B. hispida* Cogn. seed (DGZ, Lot: 3146497), *P. persica* (L.) Batsch. seed (TR, Lot: 1187553), *Coix lacryma‐jobi var. ma‐yuen* (Rom.Caill.) Stapf. seed (YYR, Lot: 1001325) were provided by Guangdong Yifang Pharmaceutical Co., Ltd. The voucher specimen (NO. J2023D0804) has been deposited at sample chamber, College of Pharmacy, Jinan University, Guangzhou, China.

LG (30 g), DGZ (30 g), TR (10 g), and YYR (20 g) were weighed, pulverized, and thoroughly mixed. The mixture was soaked in 10 volumes of water for 60 min, followed by decoction at 100°C for 90 min. After filtration, the residue was subjected to a second decoction with 8 volumes of water. The two filtrates were combined, concentrated, and lyophilized to yield 12 g of QJWJT lyophilized powder [39, 40]. The voucher specimen (NO.J2023D0804) has been deposited at sample chamber, College of Pharmacy, Jinan University, Guangzhou, China.

#### 5.1.2 Preparation of QJWJTG

LG granule (Lot: AB451301), DGZ granule (Lot: A307A821), TR granule (Lot: AB440911), and YYR granule (Lot: AB431461) were provided by Guangdong Yifang Pharmaceutical Co., Ltd. According to the compatibility ratio of QJWJTD, appropriate amounts of each granule were accurately weighed.

#### 5.1.3 Determination of Dosage Regimens

The clinical dose of QJWJT is 0.75 g of crude drug/kg and the body surface area factor is 9.1, yielding an equivalent mouse dose of approximately 7 g of crude drug/kg. The experimental design included three dose levels: a low dose of 3.5 g/kg/d, a medium dose of 7 g/kg/d, and a high dose of 14 g/kg/d [40].

### 5.2 Reagents

CCK‐8, SYBR green PCR kit and Hifair III first Strand cDNA Synthesis SuperMix were obtained from Yeasen (Shanghai, China). The NO assay kit was obtained from Beyotime (Shanghai, China). The RNA Extraction Kit was provided by Feijie Biotechnology (Shanghai, China). Blood analyzer reagents were purchased from Yuechuang (Shandong, China). The ELISA kits (TNF‐α/IL‐6/IL‐1β/IFN‐γ) were obtained from Lianke (Hangzhou, China). Anti‐HIF‐1α antibody (ab179483), anti‐respiratory syncytial virus fusion (F) glycoprotein antibody (ab24011), and goat anti‐mouse IgG H&L (Alexa Fluor 488) antibody (ab150077) were supplied by Abcam (Cambridge, UK). Horse radish peroxidase (HRP)‐conjugated anti‐mouse IgG antibody (7076) and HRP‐conjugated anti‐rabbit IgG antibody (7074) were sourced from Cell Signaling Technology (Danvers, MA, USA).

### 5.3 Cells and Virus

The RSV A2 strain (ATCC VR‐1540) was provided by the Medicinal Virology Institute of Wuhan University (Wuhan, China). Hep‐2 cells (human laryngeal carcinoma epithelial cells; ATCC CCL‐23; RRID: CVCL_0306) and RAW264.7 cells (murine macrophage cell line, ATCC TIB‐71; RRID: CVCL_0493) were obtained from the American Type Culture Collection (ATCC, Manassas, VA, USA). Growth medium consisted of DMEM plus 10% FBS and 100 U/mL PS. HEp‐2 cells served as the host for viral amplification, after which plaque assay was employed to determine titers, and the virus was kept at −80°C.

### 5.4 Cell Viability Assay

Cell viability was assessed using the CCK‐8 assay. Hep‐2 or RAW264.7 cells in logarithmic growth phase were seeded into 96‐well plates at an appropriate density. Following a 12‐h culture period, cells received QJWJTD, QJWJTG, or individual compounds at different concentrations for 24 h. Subsequently, a 10% CCK‐8 solution was introduced. Optical density (OD) was recorded at 450 nm. Raw OD values were converted to percentages, and the concentrations were logarithmically transformed before being used for statistical analysis.

### 5.5 Nitric Oxide (NO) Assay

NO production was assessed with a NO assay kit, with the operation procedure per the manufacturer's guidelines. Briefly, after being cultured for 12 h in 96‐well plates, RAW264.7 cells were subjected to RSV infection (MOI = 1) and treated with QJWJTD, QJWJTG, or individual compounds for 24 h. A 1:1 mixture of the supernatant and Griess reagent was prepared, followed by measurement of the absorbance at 540 nm.

### 5.6 RT‐qPCR Assay

RNA was isolated (FastPure Kit), reverse‐transcribed to cDNA (PrimeScript RT), and amplified by qPCR (TB Green Premix Ex Taq, TaKaRa) on a Roche system following the manufacturer's instructions. Date were normalized to the model group following correction with internal reference genes. Primers are listed in Table S5.

### 5.7 Acute Toxicity Test of QJWJT in Mice

Female BALB/c mice (4 weeks old) were randomly divided into seven groups (n = 6 per group), including a normal saline control group, QJWJTD low/mid/high dose groups (3.5, 7, 14 g/kg), and QJWJTG low/mid/high dose groups (3.5, 7, 14 g/kg). Mice in the treatment groups received the corresponding doses of QJWJTD or QJWJTG by oral gavage once daily for 7 consecutive days, while the control group was given an equal volume of normal saline. Body weight was recorded and general behavioral observations were performed daily prior to each administration throughout the experimental period.

### 5.8 Animal Experiments

Female BALB/c mice (4 weeks old) were sourced from the Guangdong Medical Laboratory Animal Center and housed under standardized conditions at 22°C with alternating 12‐h light and dark periods. Random allocation yielded nine groups of mice: normal control (uninfected), RSV‐infected control, QJWJTD (3.5, 7, 14 g/kg/d), QJWJTG (3.5, 7, 14 g/kg/d) and ribavirin (40 mg/kg/d) groups, six mice per group (n = 6). Mice were intragastrically administered with the corresponding test drugs for 5 consecutive days. On the third day of administration, the mice were anesthetized and intranasally inoculated with 100 µL of RSV (1 × 10^6^ plaque‐forming units, PFU). On the third day post‐RSV infection, mice were humanely sacrificed, followed by the collection of blood samples and sterile isolation of lung tissues. These lung tissues were then utilized for histopathological analysis, ELISA and RT‐PCR assays. Blood samples were collected to detect changes in the number of immune cells in peripheral blood, in order to evaluate the regulatory effect of QJWJT on the inflammatory response induced by RSV infection. Animal procedures strictly adhered to the Laboratory Animal Use and Care Guidelines (Chinese CDC) along with the Medical Laboratory Animal Rules (Ministry of Health, China). Ethical approval was granted by the Ethics Committees at Jinan University and the National Institute for Communicable Disease Control and Prevention (approval No. 20220314‐14).

### 5.9 UHPLC‐Q‐Orbitrap HRMS Analysis

QJWJTD or QJWJTG was dissolved in 75% ethanol, sonicated for 1 h, processed by centrifugal clarification (13000 rpm, 15 min) and membrane sterilization (0.22 µm). Mice were orally administered QJWJTD or QJWJTG (14 g/kg/day) for 3 consecutive days. Plasma was collected under anesthesia 4 h after the last administration. A threefold volume of acetonitrile was added to the plasma samples, followed by centrifugation at 13000 rpm to collect the supernatant. The supernatant was vacuum‐concentrated, reconstituted in 50% chromatographic methanol and filtered using a membrane with 0.22 µm pore size.

UHPLC‐Q‐Orbitrap HRMS (Thermo Fisher Scientific) analysis used a BEH C18 column (2.1 × 150 mm, 30°C) with a water‐methanol mobile phase at 0.2 mL/min and 8 µL injection. Gradient elution: for QJWJTD/QJWJTG, initial 5% B (0–5 min), linear gradient to 95% B (5–30 min), and 100% B (30–60 min); for blood samples, initial 5% B (0–5 min), linear gradient to 95% B (5–30 min), and 100% B (30–35 min). Electrospray ionization mass spectrometry (m/z 100–1500) used a capillary temperature of 320°C, sheath gas flow of 35 arb, and a spray current limit of 100 µA. Positive and negative ion modes employed spray voltages of 3.5 kV and 2.8 kV with auxiliary gas settings of 12 arb and 10 arb, respectively. Xcalibur software handled data analysis.

### 5.10 Quantitative Determination of Components

Gallic acid reference substance was precisely weighed and dissolved in 75% ethanol to prepare a series of reference solutions at 1, 5, 10, 20, and 50 µg/mL. QJWJTD (120 mg) and QJWJTD (130 mg) were precisely weighed, ultrasonically dissolved in 1 mL of 75% ethanol, replenished to weight, and adjusted to 1 mL. An aliquot of 0.1 mL was taken, diluted to 1.5 mL, mixed, and filtered through a 0.22 µm microporous membrane.

Isoliquiritigenin reference substance was precisely weighed and dissolved in methanol to prepare a series of reference solutions at 1, 5, 10, 20, and 40 µg/mL. QJWJT decoction (50 mg) and granules (130 mg) were precisely weighed, ultrasonically dissolved in 1 mL of methanol, replenished to weight, and adjusted to 1 mL. Aliquots of 0.2 mL and 0.1 mL were precisely taken, diluted to 1.5 mL, respectively, mixed, and filtered through a 0.22 µm microporous membrane.

Chromatographic separation was performed on an Agilent 1200‐1260 system equipped with an ULTIMATE XB‐C18 column (4.6 mm × 250 mm, 5 µm). The mobile phase consisted of 0.1% formic acid in water (A) and methanol (B) under gradient elution (0‐30 min, 10% B; 30 ‐ 40 min, 100% B; 40 ‐ 45 min, 10% B). The flow rate was 1.0 mL/min, the injection volume was 10 µL, and the detection wavelength was set at 254 nm.

### 5.11 Target Network Analysis

SMILES structures from PubChem, potential targets from SwissTargetPrediction, SEA, and STITCH, and RSV‐related targets from GeneCards, OMIM, DrugBank, DisGeNet, and TTD (using “respiratory syncytial virus pneumonia”) were obtained. After removing duplicates, the targets were consolidated to establish an RSV target set. Potential QJWJT targets against RSV were further identified using the EVenn platform. PPI data for these targets were obtained from STRING 12.0 and visualized via Cytoscape 3.10.0. Key targets were screened based on topological parameters, retaining those with Degree, CC, and BC values exceeding twice the median, thereby obtaining the core targets. Pathway enrichment (KEGG) and functional annotation (GO) were conducted using Metascape database.

### 5.12 Plaque Assay

One day before infection, Hep‐2 cells in 24‐well plates were incubated at 37°C for 2 h with diluted virus, drug‐treated virus dilution, or mouse lung viral supernatant. The washed monolayer was overlaid with 1.2% agarose medium and incubated for 4 days. After 2 h of fixation using 4% formalin, the solidified agarose layer was discarded. Cells were then exposed to 1% crystal violet for 30 min, followed by plaque counting.

### 5.13 Immunofluorescence Assay

Twenty‐four hours before infection, Hep‐2 cells were placed into 96‐well plates and incubated at 37°C for 2 h with either virus alone or virus combined with different drug concentrations. Once complete CPE was observed in cells of the RSV model group, the following treatments were performed: Fixation (4% paraformaldehyde, 60 min), permeabilization step (0.1% Triton X‐100, 10 min). Cells were then probed with primary antibodies (4°C, overnight). Afterward, fluorescent secondary antibodies were added (RT, dark, 1 h), followed by DAPI nuclear staining. Three washes with buffer were performed between each step. Images were captured using a fluorescence microscope (Carl Zeiss AG, Oberkochen, Germany).

### 5.14 H&E Staining and Histopathological Scoring of Lung Injury

Lung tissues from sacrificed mice were washed in pre‐chilled PBS, placed in 10% neutral‐buffered formalin for one day, dehydrated through graded ethanol solutions, embedded in paraffin blocks, sectioned, mounted, deparaffinized, rehydrated, and processed for histological and immunohistochemical evaluation. Bronchitis/bronchiolitis severity and hemorrhage were graded on a 0‐4 point system (graded as 0 for absent, 1 for mild, 2 for moderate, 3 for moderately severe, and 4 for severe) [21].

### 5.15 Analysis of Leukocyte Changes in Peripheral Blood

Orbital blood samples collected on day 3 post‐infection were placed into anticoagulant tubes for determination of monocyte, lymphocyte, neutrophil, and leukocyte counts using an automatic hematology analyzer [22].

### 5.16 Western Blotting Assay

Cells were lysed under ice‐cold conditions using a lysis buffer containing protease and phosphatase inhibitors for 0.5 h. After lysis, the mixture was centrifuged at 12,000 rpm for 15 min, and the supernatant was collected. Protein concentrations were determined using the BCA method, and all samples were adjusted to equal amounts. After SDS‐PAGE separation of equal protein amounts, wet transfer onto PVDF membranes was performed. Post‐transfer, membranes were blocked with 5% BSA (RT, 1 h), then incubated with primary antibodies (4°C, overnight). After TBST washes, secondary antibody incubation (RT, 2 h) was performed, followed by three further TBST washes. Finally, chemiluminescent substrate was applied, and images were captured using the Amersham Imager 600 system and quantitatively analyzed using Image J software. Data were normalized to the model group as the reference baseline.

### 5.17 Time‐of‐Addition Assay

HEp‐2 cells were seeded in 96‐well plates and grown to confluence. Cells were then infected with RSV at an MOI of 1 in 50 µL of medium, and this time point was defined as 0 h post‐infection (hpi). At 2 hpi, the inoculum was removed, and the cells were washed with PBS to eliminate unbound virus. QJWJTD (2 mg/mL) or QJWJTG (2 mg/mL) was added at the indicated times (0 hpi, co‐administered with the virus; 1, 2, 6, 10, and 18 hpi) and maintained until 24 hpi. Cell and virus control groups were included. At 24 hpi, cells were fixed with 4% paraformaldehyde for 30 min, permeabilized with 0.1% Triton X‐100 for 10 min, and blocked with 5% BSA for 60 min, with three PBS washes after each step. Subsequently, cells were incubated with mouse anti‐RSV F antibody (1:500) overnight at 4°C, washed, and then incubated with Alexa Fluor 488‐conjugated secondary antibody (1:500) for 2 h at room temperature in the dark. After washing, fluorescence intensity was measured using a microplate reader, and images were acquired with a fluorescence microscope.

### 5.18 Molecular Docking

The three‐dimensional (3D) structures of target proteins were downloaded from the Protein Data Bank (PDB; https://www.rcsb.org), and the chemical structures of small‐molecule compounds were obtained from the PubChem database (https://pubchem.ncbi.nlm.nih.gov). All structures were energy‐minimized using Discovery Studio 2025 and saved in PDB format. PDBQT files for proteins and ligands were prepared using AutoDock 1.5.6, and molecular docking was performed with AutoDock Vina 1.2.5. The docking results were visualized and analyzed using PyMOL 3.1.8 and Discovery Studio 2025.

### 5.19 Statistical Analysis

Each assay was repeated a minimum of three times. Data are mean ± SD (GraphPad Prism 9.1). Statistical significance was determined by one‐way ANOVA followed by Tukey's post hoc test for multiple comparisons, with *p* < 0.05 considered significant.

## Author Contribution Statement

**Chu‐Yi Liao**: Writing – original draft, Investigation, Data curation, Software. **Mei‐Lan Yang**: Writing – review and editing, Data curation. **Ze‐Ping Chen**: Data Curation; **Bin Chen**: Writing – review and editing, Formal analysis. **Yu‐Jun Tang**: Project administration, Data curation. **Zhen Wang**: Software. **Hui‐Nan Zhao**: Visualization. **Su‐Hua Deng**: Supervision. **Jin‐Hai Ye**: Supervision. **Zhong Liu**: Writing – review and editing, Methodology, Funding acquisition. **Li‐Feng Chen**: Conceptualization, Methodology. **Man‐Mei Li**: Methodology, Project administration, Resources, Validation.

## Conflicts of Interest

The authors declare no conflicts of interest.

## Supporting information




**Supporting File**: cbdv71731‐sup‐0001‐SuppMat.docx

## Data Availability

The data that support the findings of this study are available from the corresponding author upon reasonable request.
