# Supplementary material for: Qianjinweijingtang Decoction and Its Granule Inhibit Respiratory Syncytial Virus Infection and the Associated Inflammatory Response
Source: Chem Biodivers. 2026 Sep 20;23(9):e71731. doi: 10.1002/cbdv.71731 (PMC13590134; doi:10.1002/cbdv.71731)
Supplement: Supplementary file 1 [file CBDV-23-e71731-s001.docx]

**Supporting Information**

**Contents**

[Figure. S1 Time-of-addition assay	1](#_Toc22861)

[Figure. S2 Quantitative analysis	2](#_Toc27270)

[Table. S1 Characterization of chemical compounds in QJWJTD	3](#_Toc11244)

[Table. S2 Characterization of chemical compounds in QJWJTG	8](#_Toc13240)

[Table. S3 Absorbed components of QJWJTD in mouse plasma	16](#_Toc30414)

[Table. S4 Absorbed components of QJWJTG in mouse plasma	19](#_Toc8910)

[Table. S5 Primer sequences	22](#_Toc28915)

**Figure. S1 Time-of-addition assay**

Figure. S1 Effect of QJWJT on RSV entry. **(a-d)** HEp-2 cells were inoculated with RSV A2 (MOI = 1) and exposed to QJWJTD (2 mg/mL) or QJWJTG (2 mg/mL) at 0, 1, 2, 6, 12, and 18 h post-inoculation. Following a 24-h incubation period, the cells were labeled with anti-RSV-F antibodies, and fluorescence signals were quantified using fluorescence microscopy. Data are presented as mean ± SD (n = 3).

**Figure. S2 Quantitative analysis**

Figure.S2 Quantitative analysis. **(a-b)** Standard curves of isoliquiritigenin and gallic acid. **(c)** The contents of isoliquiritigenin and gallic acid in QJWJT decoction and granules were determined by the external standard method.

**Table. S1 Characterization of chemical compounds in QJWJTD**

| **NO.** | **RT (min)** | **Formula** | **Adduct** | **m/z** | **Error (ppm)** | **MS/MS** | **Compounds** | **Structure type** | **Source** |
| --- | --- | --- | --- | --- | --- | --- | --- | --- | --- |
| 1 | 1.09 | C_7_H_12_O_6_ | [M-H]-1 | 191.0550 | -2.9 | 173.0445，127.0389，85.0282 | Quinic acid | Organic acids | TR |
| 2 | 1.10 | C_5_H_9_NO_4_ | [M-H]-1 | 146.0446 | -5.0 | 128.0344，102.0546 | Glutamic acid | Amino Acids and Derivatives | TR，DGZ |
| 3 | 1.12 | C_6_H_14_N_4_O_2_ | [M+H]+1 | 175.1196 | 0.6 | 130.0972，116.0705，70.0656，60.0562 | Arginine | Amino Acids and Derivatives | DGZ |
| 4 | 1.12 | C_5_H_10_O_5_ | [M-H]-1 | 149.0443 | -4.7 | 89.0231，75.0074，59.0125 | Xylose | Saccharides | YYR，LG |
| 5 | 1.14 | C_6_H_13_N_3_O_3_ | [M-H]-1 | 174.0873 | -3.3 | 159.0304，141.0000，70.6782 | Citrulline | Amino Acids and Derivatives | DGZ |
| 6 | 1.15 | C_6_H_14_O_6_ | [M-H]-1 | 181.0706 | -3.4 | 101.0231，89.0230，71.0125，59.0125 | Mannitol | Saccharides | DGZ |
| 7 | 1.16 | C_5_H_11_NO_2_ | [M+H]+1 | 118.0869 | 0.8 | 105.0456，95.0497，70.0393 | Betaine | Alkaloids | LG |
| 8 | 1.21 | C_7_H_7_NO_2_ | [M+H]+1 | 138.0556 | 0.7 | 120.0446，94.0401 | Trigonelline | Alkaloids | DGZ |
| 9 | 1.40 | C_6_H_11_NO_2_ | [M+H]+1 | 130.0869 | 0.7 | 84.0813，84.0451，74.0972 | Pipecolic acid | Others | LG |
| 10 | 1.40 | C_8_H_8_O_4_ | [M-H]-1 | 167.0337 | -4.4 | 152.0104，123.0411，108.0205 | vanillic acid | Phenolic Acids | YYR，TR，LG |
| 11 | 1.54 | C_9_H_12_N_2_O_6_ | [M-H]-1 | 243.0620 | 1.2 | 200.0563，152.0344，140.0348 | Uridine | Alkaloids | TR |
| 12 | 1.61 | C_6_H_8_O_7_ | [M-H]-1 | 191.0193 | 0.6 | 129.0802，111.0081，85.0278 | Citric acid | Others | TR |
| 13 | 1.63 | C_19_H_26_O_13_ | [M-H]-1 | 461.1293 | -0.5 | 281.0669，113.0235 | Sibiricose A3 | Saccharides | YYR |
| 14 | 1.66 | C_7_H_6_O_4_ | [M-H]-1 | 153.0183 | -3.2 | 109.0283，81.0333 | Protocatechuic acid | Phenolic Acids | YYR |
| 15 | 1.69 | C_4_H_6_O_5_ | [M-H]-1 | 133.0130 | -4.2 | 115.0023，88.0472，87.0438 | Malic acid | Organic acids | TR |
| 16 | 1.81 | C_9_H_11_NO_3_ | [M-H]-1 | 180.0657 | -2.0 | 163.0391，119.0489，93.0334 | Tyrosine | Amino Acids and Derivatives | YYR |
| 17 | 1.99 | C_7_H_6_O_5_ | [M-H]-1 | 169.0138 | 0.6 | 125.0236，97.0286，81.0329 | Gallic acid | Phenolic Acids | YYR |

**Table. S1 Characterization of chemical compounds in QJWJTD (continued)**

| **NO.** | **RT (min)** | **Formula** | **Adduct** | **m/z** | **Error (ppm)** | **MS/MS** | **Compounds** | **Structure type** | **Source** |
| --- | --- | --- | --- | --- | --- | --- | --- | --- | --- |
| 18 | 2.85 | C_8_H_7_NO_3_ | [M+H]+1 | 166.0506 | 1.1 | 148.0397，138.0551，123.0441 | Coixol | Others | LG，YYR |
| 19 | 3.36 | C_16_H_18_O_9_ | [M-H]-1 | 353.0869 | -1.0 | 191.0551，179.0340，135.0439 | Chlorogenic acid | Phenolic Acids | TR，YYR |
| 20 | 6.01 | C_8_H_8_O_3_ | [M-H]-1 | 151.0388 | -4.8 | 107.0127，83.0126，65.0021 | 4-Hydroxyphenylacetic acid | Organic acids | TR |
| 21 | 6.51 | C_10_H_13_N_5_O_4_ | [M+H]+1 | 268.1032 | -4.1 | 136.0615，85.0283 | Adenosine | Alkaloids | YYR |
| 22 | 6.54 | C_5_H_5_N_5_ | [M-H]-1 | 134.0460 | -5.0 | 119.0351，94.04011 | Adenine | Alkaloids | YYR |
| 23 | 7.60 | C_9_H_10_O_4_ | [M-H]-1 | 181.0496 | -2.7 | 137.0595，122.0362，121.0275 | Homovanillic Acid | Phenolic Acids | LG |
| 24 | 7.81 | C_7_H_6_O | [M+H]+1 | 107.0500 | 3.2 | 105.0652，79.0546 | Benzaldehyde | Aldehydes | TR |
| 25 | 7.83 | C_9_H_10_O_5_ | [M-H]-1 | 197.0444 | -3.0 | 182.0209，153.0545，138.0310 | Syringic acid | Phenolic Acids | YYR |
| 26 | 8.06 | C_6_H_11_NO | [M+H]+1 | 114.0924 | 4.5 | 96.0809，85.0652，69.0704 | Caprolactam | Others | YYR |
| 27 | 8.82 | C_12_H_12_N_2_O_2_ | [M+H]+1 | 217.0987 | 4.6 | 171.0911，144.0807，130.0650 | 1,2,3,4-Tetrahydro-beta-carboline-3-carboxylic acid | Amino Acids and Derivatives | YYR |
| 28 | 9.20 | C_10_H_10_O_3_ | [M+H]+1 | 179.0718 | 0.4 | 161.0595，147.0438，119.0491 | Coniferyl aldehyde | Aldehydes | TR |
| 29 | 9.67 | C_9_H_6_O_4_ | [M+H]+1 | 179.0356 | -0.4 | 164.9838，147.0436 | Esculetin | Others | TR，YYR |
| 30 | 9.85 | C_7_H_6_O_2_ | [M+H]+1 | 123.0450 | 3.2 | 95.0493，77.0389 | 4-Hydroxybenzaldehyde | Aldehydes | YYR |
| 31 | 10.41 | C_9_H_8_O_3_ | [M-H]-1 | 163.0401 | 3.4 | 145.0287，135.0492，119.0491 | p-Coumaric acid | Phenolic Acids | TR，YYR，LG |

**Table. S1 Characterization of chemical compounds in QJWJTD (continued)**

| **NO.** | **RT (min)** | **Formula** | **Adduct** | **m/z** | **Error (ppm)** | **MS/MS** | **Compounds** | **Structure type** | **Source** |
| --- | --- | --- | --- | --- | --- | --- | --- | --- | --- |
| 32 | 10.78 | C_19_H_22_O_6_ | [M-H]-1 | 345.1336 | -0.6 | 257.1541，239.1432 | Gibberellic acid | Terpenoids and Steroids | TR |
| 33 | 10.94 | C_14_H_17_NO_6_ | [M+H]+1 | 296.1127 | -2.4 | 132.0454，105.342，77.0394 | Prunasin | Saccharides | TR |
| 34 | 11.39 | C_15_H_14_O_6_ | [M-H]-1 | 289.0711 | -0.4 | 245.0814，203.0705，109.0284 | catechin | Flavonoids | TR，YYR |
| 35 | 12.00 | C_11_H_12_O_5_ | [M-H]-1 | 223.0602 | -2.0 | 208.0366，164.0467，149.0231 | Sinapinic acid | Phenolic Acids | YYR |
| 36 | 12.37 | C_10_H_12_O_3_ | [M-H]-1 | 179.0703 | -2.9 | 163.0753，153.0293，151.0389 | Coniferyl alcohol | Terpenoids and Steroids | YYR |
| 37 | 12.59 | C_17_H_20_N_4_O_6_ | [M+H]+1 | 377.1475 | 3.7 | 359.1344，243.0875， 117.05481, | Riboflavin | Others | LG |
| 38 | 12.86 | C_22_H_26_O_8_ | [M-H]-1 | 417.1544 | -1.3 | 343.1177.153.0545 | Syringaresinol | Others | YYR |
| 39 | 13.06 | C_10_H_10_O_4_ | [M-H]-1 | 193.0497 | -2.0 | 178.0495，149.0595，134.0360 | Ferulic acid | Phenolic Acids | YYR，TR，LG |
| 40 | 15.29 | C_20_H_27_NO_11_ | [M-H]-1 | 456.1502 | -0.8 | 145.0495，85.0284 | Amygdalin | Saccharides | TR |
| 41 | 16.00 | C_27_H_30_O_16_ | [M-H]-1 | 609.1449 | -1.1 | 302.0369，300.0268，151.0025 | Rutin | Saccharides | DGZ，YYR |
| 42 | 16.06 | C_20_H_20_O_7_ | [M+H]+1 | 373.1274 | -3.6 | 358.1045，343.0811，325.0709 | Tangeretin | Flavonoids | YYR |
| 43 | 16.67 | C_28_H_34_O_15_ | [M-H]-1 | 609.1814 | -0.9 | 300.0268，301.0338，151.0025 | Hesperidin | Saccharides | YYR |
| 44 | 16.89 | C_10_H_8_O_4_ | [M+H]+1 | 193.0503 | 1.1 | 178.0264，149.0596，133.0284 | Scopoletin | Others | TR |
| 45 | 16.94 | C_16_H_14_O_6_ | [M-H]-1 | 301.0709 | -1.0 | 286.0475，171.1014 | Hesperetin | Flavonoids | TR |
| 46 | 17.99 | C_15_H_12_O_6_ | [M-H]-1 | 287.0555 | -0.2 | 243.0654，174.0914 | Eriodictyol | Flavonoids | TR，YYR |

**Table. S1 Characterization of chemical compounds in QJWJTD (continued)**

| **NO.** | **RT (min)** | **Formula** | **Adduct** | **m/z** | **Error (ppm)** | **MS/MS** | **Compounds** | **Structure type** | **Source** |
| --- | --- | --- | --- | --- | --- | --- | --- | --- | --- |
| 47 | 18.42 | C_17_H_14_O_7_ | [M+H]+1 | 331.0803 | -4.5 | 313.0708，285.0778，245.0813 | Aflatoxin G2 | Others | TR |
| 48 | 19.50 | C_15_H_10_O_7_ | [M-H]-1 | 301.0346 | -0.8 | 178.9974，151.0024 | Quercetin | Flavonoids | DGZ，YYR，TR |
| 49 | 19.52 | C_15_H_14_O_5_ | [M+H]+1 | 275.0908 | -4.2 | 275.0907，169.0635，151.0386 | Phloretin | Flavonoids | YYR |
| 50 | 19.80 | C_15_H_12_O_5_ | [M-H]-1 | 271.0607 | 0.2 | 177.0181，151.0024 | Naringenin | Flavonoids | TR |
| 51 | 20.18 | C_16_H_12_O_7_ | [M-H]-1 | 315.0502 | -0.9 | 300.0273，253.0895，209.1917 | Isorhamnetin | Flavonoids | DGZ |
| 52 | 20.46 | C_16_H_12_O_4_ | [M+H]+1 | 269.0806 | -2.9 | 212.9725，1922.9611，148.9716 | Formononetin | Flavonoids | YYR |
| 53 | 20.78 | C_10_H_8_O_5_ | [M-H]-1 | 207.0295 | 0.7 | 192.0052，147.0078，120.0222 | Fraxetin | Flavonoids | LG |
| 54 | 20.82 | C_11_H_20_O_3_ | [M-H]-1 | 199.1329 | -2.6 | 181.1222，155.1429，127.1116 | (S)-9-Hydroxy-10-undecenoic acid | Fatty Acids | YYR |
| 55 | 22.10 | C_10_H_16_O | [M+H]+1 | 153.1280 | 0.4 | 135.1168，125.1322 | Camphor | Terpenoids and Steroids | LG |
| 56 | 23.01 | C_18_H_22_O_5_ | [M-H]-1 | 317.1396 | 2.2 | 273.1558，175.0441，131.0530 | Zearalenone | Others | TR |
| 57 | 23.81 | C_15_H_12_O_4_ | [M-H]-1 | 255.0656 | -0.5 | 125.0959，101.0596，67.4514 | Isoliquiritigenin | Flavonoids | YYR |
| 58 | 27.92 | C_24_H_38_O_4_ | [M-H]-1 | 389.2687 | -1.2 | 279.2312，247.1685 | Dioctyl phthalate | Esters | YYR |
| 59 | 30.14 | C_18_H_30_O_2_ | [M+H]+1 | 279.2309 | 3.9 | 261.2203，123.1167 | α-Linolenic acid | Fatty Acids | YYR，DGZ，TR，LG |
| 60 | 30.99 | C_29_H_50_O | [M+H]+1 | 415.3924 | -3.7 | 397.3829，215.2682，297.2577 | beita-Sitosterol | Terpenoids and Steroids | TR，YYR，LG，DGZ |
| 61 | 31.13 | C_18_H_34_O_3_ | [M+H]+1 | 299.2574 | -4.1 | 279.2321，183.1380 | 12-Hydroxy-9-octadecenoic acid | Fatty Acids | DGZ |

**Table. S1 Characterization of chemical compounds in QJWJTD (continued)**

| **NO.** | **RT (min)** | **Formula** | **Adduct** | **m/z** | **Error (ppm)** | **MS/MS** | **Compounds** | **Structure type** | | **Source** |
| --- | --- | --- | --- | --- | --- | --- | --- | --- | --- | --- |
| 62 | 31.48 | C_18_H_34_O_2_ | [M+H]+1 | 283.2636 | -0.4 | 129.1523，98.0074 | Elaidic Acid | Fatty Acids | TR，YYR | |
| 63 | 31.90 | C_16_H_30_O_2_ | [M+H]+1 | 255.2311 | 4.3 | 142.3847，67.0837 | Palmitoleic acid | Fatty Acids | YYR | |
| 64 | 32.51 | C_18_H_32_O_3_ | [M-H]-1 | 295.2281 | 2.6 | 277.2165，195.1378，171.1014 | 13-HODE | Fatty Acids | YYR | |
| 65 | 35.01 | C_14_H_28_O_2_ | [M-H]-1 | 227.2006 | -2.2 | 99.7051，75.4008 | Myristic acid | Fatty Acids | YYR | |
| 66 | 37.32 | C_30_H_48_O_3_ | [M-H]-1 | 455.3520 | -1.1 | 305.2480，237.2379，261.2582 | Oleanolic acid | Organic acids | LG | |
| 67 | 37.82 | C_20_H_38_O_2_ | [M+H]+1 | 311.2936 | -4.5 | 311.2941，265.2522，237.2555 | Ethyl oleate | Esters | YYR | |
| 68 | 43.92 | C_15_H_30_O_2_ | [M-H]-1 | 241.2165 | -1.1 | 223.2607，197.2275，59.0138 | Pentadecanoic acid | Fatty Acids | YYR，DGZ | |
| 69 | 47.03 | C_20_H_34_O_2_ | [M-H]-1 | 305.2477 | -1.2 | 287.2384，261.2588 | Eicosatrienoic acid | Fatty Acids | YYR | |
| 70 | 51.28 | C_16_H_32_O_2_ | [M-H]-1 | 255.2320 | -1.6 | 255.2320，120.3208 | Hexadecanoic acid | Fatty Acids | TR，YYR，TR，LG | |
| 71 | 51.67 | C_22_H_42_O_4_ | [M+H]+1 | 371.3145 | -4.4 | 129.0543，111.0440，101.0598 | Bis(2-ethylhexyl)adipate | Esters | YYR | |
| 72 | 53.19 | C_19_H_38_O_2_ | [M-H]-1 | 297.2791 | -0.9 | 253.2536，183.0117 | Nonadecylic acid | Fatty Acids | YYR | |
| 73 | 54.37 | C_25_H_50_O_2_ | [M-H]-1 | 381.3729 | -0.9 | 363.3634，337.3839309.3512 | Pentacosanoic acid | Fatty Acids | DGZ | |
| 74 | 54.60 | C_29_H_48_O | [M+H]+1 | 413.3761 | 2.7 | 177.1630，97.1013，83.0858，57.0705 | Stigmasterol | Terpenoids and Steroids | YYR，LG，DGZ | |

**Table. S2 Characterization of chemical compounds in QJWJTG**

| **NO.** | **RT (min)** | **Formula** | **Adduct** | **m/z** | **Error (ppm)** | **MS/MS** | **Compounds** | **Structure type** | **Source** |
| --- | --- | --- | --- | --- | --- | --- | --- | --- | --- |
| 1 | 1.09 | C_4_H_6_O_5_ | [M-H]-1 | 133.0140 | 2.3 | 115.0023，88.0471，87.0438 | Malic acid | Aldehydes | TR |
| 2 | 1.09 | C_7_H_12_O_6_ | [M-H]-1 | 191.0550 | -2.9 | 173.0443，127.0387，85.0282 | Quinic acid | Others | YYR |
| 3 | 1.10 | C_6_H_12_O_6_ | [M-H]-1 | 179.0551 | -2.6 | 161.0479，142.0345 | Fructose | Amino Acids and Derivatives | DGZ |
| 4 | 1.12 | C_6_H_14_N_4_O_2_ | [M+H]+1 | 175.1196 | 3.7 | 130.0975，116.0709，70.0645 | Arginine | Aldehydes | YYR |
| 5 | 1.12 | C_5_H_10_O_5_ | [M-H]-1 | 149.0453 | 2.0 | 89.0234，75.0079，59.0126 | Xylose | Alkaloids | DGZ |
| 6 | 1.14 | C_6_H_13_N_3_O_3_ | [M-H]-1 | 174.0873 | -3.3 | 159.0306，141.0005，70.6784 | Citrulline | Others | LG |
| 7 | 1.15 | C_6_H_14_O_6_ | [M-H]-1 | 181.0706 | -3.4 | 101.0235，89.0238，59.0125 | Mannitol | Amino Acids and Derivatives | YYR，DGZ |
| 8 | 1.16 | C_5_H_11_NO_2_ | [M+H]+1 | 118.0861 | -1.3 | 105.0457，95.0499，70.0386 | Betaine | Organic acids | TR |
| 9 | 1.21 | C_5_H_9_NO_2_ | [M+H]+1 | 116.0705 | 3.8 | 70.0655，68.0530 | proline | Alkaloids | YYR |
| 10 | 1.21 | C_7_H_7_NO_2_ | [M+H]+1 | 138.0546 | -2.6 | 120.0446，94.0405 | Trigonelline | Alkaloids | DGZ |
| 11 | 1.30 | C_5_H_11_NO_2_ | [M+H]+1 | 118.0861 | -1.3 | 72.0823，57.0603 | Valine | Phenolic Acids | YYR，TR |
| 12 | 1.40 | C_6_H_8_O_7_ | [M-H]-1 | 191.0187 | -2.5 | 129.0189，111.0075，87.0075，85.0282 | Citric acid | Fatty Acids | YYR |
| 13 | 1.40 | C_6_H_11_NO_2_ | [M+H]+1 | 130.0861 | -1.2 | 84.0013，74.0972 | Pipecolic acid | Phenolic Acids | TR |
| 14 | 1.54 | C_9_H_12_N_2_O_6_ | [M-H]-1 | 243.0620 | 1.2 | 200.0567，152.0346，140.0349 | uridine | Alkaloids | TR |
| 15 | 1.56 | C_6_H_13_NO_2_ | [M+H]+1 | 132.1017 | 2.6 | 86.0963，69.0704 | Leucine | Amino Acids and Derivatives | TR，DGZ |
| 16 | 1.65 | C_7_H_6_O_3_ | [M-H]-1 | 137.0239 | 0.2 | 94.0287，93.0334 | 4-Hydroxybenzoic acid | Saccharides | YYR，LG |

**Table. S2 Characterization of chemical compounds in QJWJTG (continued)**

| **NO.** | **RT (min)** | **Formula** | **Adduct** | **m/z** | **Error (ppm)** | **MS/MS** | **Compounds** | **Structure type** | **Source** |
| --- | --- | --- | --- | --- | --- | --- | --- | --- | --- |
| 17 | 1.66 | C_7_H_6_O_4_ | [M-H]-1 | 153.0193 | 3.4 | 109.0283，81.0333 | Protocatechuic acid | Aldehydes | YYR |
| 18 | 1.81 | C_9_H_11_NO_3_ | [M-H]-1 | 180.0657 | -2.0 | 119.0486，93.0353 | Tyrosine | Phenolic Acids | LG |
| 19 | 1.99 | C_7_H_6_O_5_ | [M-H]-1 | 169.0138 | 0.6 | 125.0236，97.0286，81.0329 | Gallic acid | Phenolic Acids | YYR |
| 20 | 2.41 | C_7_H_6_O_5_ | [M-H]-1 | 169.0132 | -2.9 | 125.02333，97.0276，81.0332 | Gallic acid | Fatty Acids | YYR |
| 21 | 2.85 | C_8_H_7_NO_3_ | [M+H]+1 | 166.0496 | -1.6 | 148.0399，138.0555，123.0446 | Coixol | Others | LG |
| 22 | 3.34 | C_19_H_28_O_11_ | [M-H]-1 | 431.1549 | -1.0 | 341.0655，311.0562 | Benzyl 6-O-beta-D-glucopyranosyl-beta-D-glucopyranoside | Phenolic Acids | YYR |
| 23 | 3.36 | C_16_H_18_O_9_ | [M-H]-1 | 353.0869 | -1.0 | 191.0556，179.0344，135.0439 | Chlorogenic acid | Aldehydes | TR |
| 24 | 3.39 | C_23_H_26_N_2_O_4_ | [M+H]+1 | 395.1978 | 4.6 | 393.1807，379.1654 | Brucine | Alkaloids | LG |
| 25 | 3.87 | C_5_H_7_N_3_O | [M+H]+1 | 126.0660 | 2.9 | 109.0285，83.0606 | 5-Methylcytosine | Amino Acids and Derivatives | DGZ |
| 26 | 5.33 | C_15_H_18_O_8_ | [M-H]-1 | 325.0918 | -1.7 | 207.0649，147.0437 | Melilotoside | Fatty Acids | YYR |
| 27 | 6.28 | C_11_H_12_N_2_O_2_ | [M+H]+1 | 205.0976 | 4.8 | 205.1216，188.0693 | Tryptophan | Others | TR，YYR |
| 28 | 6.43 | C_16_H_18_O_9_ | [M-H]-1 | 353.0869 | -1.0 | 191.0551，179.0340，135.0439 | Neochlorogenic acid | Aldehydes | TR |
| 29 | 6.51 | C_10_H_13_N_5_O_4_ | [M+H]+1 | 268.1032 | -3.1 | 136.0628，85.0283 | Adenosine | Phenolic Acids | YYR，LG |

**Table. S2 Characterization of chemical compounds in QJWJTG (continued)**

| **NO.** | **RT (min)** | **Formula** | **Adduct** | **m/z** | **Error (ppm)** | **MS/MS** | **Compounds** | **Structure type** | **Source** |
| --- | --- | --- | --- | --- | --- | --- | --- | --- | --- |
| 30 | 6.54 | C_5_H_5_N_5_ | [M-H]-1 | 134.0470 | 2.5 | 119.0353，94.04014 | Adenine | Saccharides | YYR |
| 31 | 7.60 | C_9_H_10_O_4_ | [M-H]-1 | 181.0496 | -2.7 | 137.0597，122.0366，121.0277 | Homovanillic Acid | Terpenoids and Steroids | YYR |
| 32 | 7.77 | C_7_H_6_O_3_ | [M+H]+1 | 139.0388 | 2.7 | 121.0285，69.0337 | Salicylic acid | Amino Acids and Derivatives | YYR |
| 33 | 7.81 | C_7_H_6_O | [M+H]+1 | 107.0493 | 1.8 | 106.0655，79.0546 | Benzaldehyde | Saccharides | DGZ |
| 34 | 8.06 | C_11_H_15_N_5_O_4_ | [M+H]+1 | 282.1193 | 0.6 | 136.0614，101.0597 | 2'-O-Methyladenosine | Organic acids | TR |
| 35 | 8.06 | C_6_H_11_NO | [M+H]+1 | 114.0914 | 0.5 | 96.0804，85.0652，69.0704 | Caprolactam | Organic acids | TR |
| 36 | 8.82 | C_12_H_12_N_2_O_2_ | [M+H]+1 | 217.0967 | -2.1 | 171.0914，144.0803，130.0655 | 1,2,3,4-Tetrahydro-beta-carboline-3-carboxylic acid | Amino Acids and Derivatives | YYR |
| 37 | 8.95 | C_7_H_6_O_4_ | [M+H]+1 | 155.0336 | 1.7 | 123.0076，109.0282，81.0347 | Gentisic acid | Phenolic Acids | TR，YYR |
| 38 | 9.20 | C_10_H_10_O_3_ | [M+H]+1 | 179.0699 | -2.1 | 179.0705，161.0596，147.0434 | Coniferyl aldehyde | Amino Acids and Derivatives | YYR |
| 39 | 9.33 | C_9_H_10_O_5_ | [M-H]-1 | 197.0445 | -2.5 | 182.0216，166.9978，153.0548 | Syringate | Fatty Acids | YYR |
| 40 | 9.66 | C_9_H_6_O_4_ | [M+H]+1 | 179.0336 | -1.6 | 164.9838，147.0433 | Esculetin | Fatty Acids | YYR，DGZ |
| 41 | 9.85 | C_7_H_6_O_2_ | [M+H]+1 | 123.0440 | -0.5 | 95.0495，77.0386 | 4-Hydroxybenzaldehyde | Fatty Acids | YYR |
| 42 | 10.35 | C_19_H_24_O_6_ | [M-H]-1 | 347.1490 | -1.3 | 329.1385，303.1595 | Gibberellin A60 | Flavonoids | YYR |

**Table. S2 Characterization of chemical compounds in QJWJTG (continued)**

| **NO.** | **RT (min)** | **Formula** | **Adduct** | **m/z** | **Error (ppm)** | **MS/MS** | **Compounds** | **Structure type** | **Source** |
| --- | --- | --- | --- | --- | --- | --- | --- | --- | --- |
| 43 | 10.41 | C_9_H_8_O_3_ | [M-H]-1 | 163.0401 | 3.4 | 145.0282，135.0486，119.0493 | p-Coumaric acid | Fatty Acids | TR，YYR，TR，LG |
| 44 | 10.94 | C_14_H_17_NO_6_ | [M+H]+1 | 296.1125 | -1.2 | 132.0453，105.342，77.0394 | Prunasin | Alkaloids | YYR |
| 45 | 11.39 | C_15_H_14_O_6_ | [M-H]-1 | 289.0711 | -0.4 | 245.0814，203.0707，109.0284 | Catechin | Flavonoids | YYR |
| 46 | 11.39 | C_15_H_14_O_6_ | [M-H]-1 | 289.0711 | -0.4 | 245.0823，203.0711 | Epicatechin | Flavonoids | TR |
| 47 | 11.47 | C_8_H_8_O_3_ | [M+H]+1 | 153.0542 | 0.8 | 125.0595，111.0441，93.0337 | Vanillin | Flavonoids | YYR |
| 48 | 11.70 | C_27_H_36_O_13_ | [M-H]-1 | 567.2070 | -1.4 | 387.1085，209.0443 | Citrusin B | Esters | DGZ |
| 49 | 12.37 | C_10_H_12_O_3_ | [M-H]-1 | 179.0703 | -2.9 | 163.0756，153.0293，151.0389 | Coniferyl alcohol | Fatty Acids | YYR，DGZ，TR，LG |
| 50 | 12.85 | C_8_H_16_O_2_ | [M-H]-1 | 143.1066 | -4.2 | 99.0802，71.0128，59.0124 | Caprylic acid | Fatty Acids | YYR，TR，DGZ |
| 51 | 12.86 | C_22_H_26_O_8_ | [M-H]-1 | 417.1544 | -1.3 | 343.1177.153.0545 | Syringaresinol | Alkaloids | YYR |
| 52 | 13.06 | C_10_H_10_O_4_ | [M-H]-1 | 193.0497 | -2.0 | 178.0495，149.0595，134.0360 | Ferulic acid | Esters | YYR |
| 53 | 14.40 | C_27_H_32_O_14_ | [M-H]-1 | 579.1707 | -1.2 | 459.1131，313.0718，271.0609，151.0025 | Naringin | Flavonoids | TR，YYR |
| 54 | 15.30 | C_20_H_27_NO_11_ | [M-H]-1 | 456.1502 | -0.8 | 145.0496，85.0277 | Amygdalin | Phenolic Acids | YYR，TR，LG |
| 55 | 15.34 | C_15_H_20_O_4_ | [M+H]+1 | 265.1423 | -2.2 | 209.1168，191.1059 | Abscisic acid | Flavonoids | TR，YYR |

**Table. S2 Characterization of chemical compounds in QJWJTG (continued)**

| **NO.** | **RT (min)** | **Formula** | **Adduct** | **m/z** | **Error (ppm)** | **MS/MS** | **Compounds** | **Structure type** | **Source** |
| --- | --- | --- | --- | --- | --- | --- | --- | --- | --- |
| 56 | 15.72 | C_15_H_12_O_5_ | [M-H]-1 | 271.0615 | 3.1 | 212.9724，194.9623，182.9625 | Naringenin | Flavonoids | TR |
| 57 | 15.76 | C_16_H_14_O_6_ | [M+H]+1 | 303.0862 | 1.4 | 284.3332，153.0188 | Hesperetin | Flavonoids | TR |
| 58 | 16 | C_27_H_30_O_16_ | [M-H]-1 | 609.1449 | -1.1 | 302.0369，300.0269，151.0025 | Rutin | Flavonoids | TR |
| 59 | 16.01 | C_21_H_20_O_12_ | [M-H]-1 | 463.0873 | -0.8 | 300.0266，178.9972 | Quercetin-3β-D-glucoside | Saccharides | TR |
| 60 | 16.06 | C_10_H_8_O_5_ | [M-H]-1 | 207.0296 | 1.2 | 192.0056，108.2193 | Fraxetin | Flavonoids | LG |
| 61 | 16.67 | C_28_H_34_O_15_ | [M-H]-1 | 609.1814 | -0.9 | 300.0264，151.0025 | Hesperidin | Fatty Acids | YYR |
| 62 | 17.33 | C_9_H_10_O_4_ | [M+H]+1 | 183.0651 | 2.5 | 140.04775，123.0456，95.0508 | Syringaldehyde | Aldehydes | LG，TR |
| 63 | 17.34 | C_16_H_12_O_5_ | [M+H]+1 | 285.0755 | 1 | 192.9612，177.9386，166.9822 | Biochanin A | Flavonoids | YYR |
| 64 | 17.73 | C_28_H_32_O_16_ | [M-H]-1 | 623.1603 | -1.5 | 300.0489，300.0268 | Isorhamnetin rutinoside | Fatty Acids | YYR |
| 65 | 17.99 | C_15_H_12_O_6_ | [M-H]-1 | 287.0555 | -0.2 | 243.0651，174.0914 | Eriodictyol | Others | DGZ |
| 66 | 18.60 | C_9_H_8_O_4_ | [M+H]+1 | 181.0492 | 1.2 | 135.0438，122.0363 | Caffeic acid | Flavonoids | DGZ，YYR，TR |
| 67 | 19.50 | C_15_H_10_O_7_ | [M-H]-1 | 301.0346 | -0.8 | 178.9974，151.0024 | Quercetin | Fatty Acids | YYR |
| 68 | 19.52 | C_15_H_14_O_5_ | [M+H]+1 | 275.0908 | -2.2 | 275.0907，169.0635，151.0386 | Phloretin | Esters | YYR |
| 69 | 19.80 | C_15_H_12_O_5_ | [M-H]-1 | 271.0607 | 0.2 | 177.0181，151.0024 | Naringenin | Flavonoids | DGZ |

**Table. S2 Characterization of chemical compounds in QJWJTG (continued)**

| **NO.** | **RT (min)** | **Formula** | **Adduct** | **m/z** | **Error (ppm)** | **MS/MS** | **Compounds** | **Structure type** | **Source** |
| --- | --- | --- | --- | --- | --- | --- | --- | --- | --- |
| 70 | 20.18 | C_16_H_12_O_7_ | [M-H]-1 | 315.0502 | -0.9 | 300.0267，271.1924 | Isorhamnetin | Flavonoids | TR |
| 71 | 20.82 | C_11_H_20_O_3_ | [M-H]-1 | 199.1329 | -2.6 | 181.1221，155.1424，127.1115 | 9-Hydroxy-10-undecenoic acid | Phenolic Acids | YYR |
| 72 | 21.28 | C_9_H_18_O_3_ | [M-H]-1 | 173.1172 | -3.3 | 127.1115，67.3914 | 9-Hydroxynonanoic acid | Esters | YYR |
| 73 | 21.42 | C_9_H_18_O_2_ | [M-H]-1 | 157.1232 | 2.2 | 139.0747，113.0959，59.0137 | Nonanoic acid | Esters | YYR |
| 74 | 21.92 | C_16_H_12_O_5_ | [M-H]-1 | 283.0614 | 2.7 | 269.0410，268.0373 | Acacetin | Flavonoids | TR |
| 75 | 22.56 | C_16_H_12_O_4_ | [M+H]+1 | 269.0797 | -4.2 | 181.0999，136.0635 | Formononetin | Esters | LG |
| 76 | 23.01 | C_18_H_22_O_5_ | [M-H]-1 | 317.1396 | 2.2 | 273.1558，175.0442，131.0532 | Zearalenone | Others | TR |
| 77 | 23.81 | C_15_H_12_O_4_ | [M-H]-1 | 255.0656 | -0.5 | 125.0959，101.0598，67.4517 | Isoliquiritigenin | Terpenoids and Steroids | YYR，LG，DGZ |
| 78 | 25.23 | C_15_H_10_O_4_ | [M-H]-1 | 253.0491 | -3.9 | 209.0605，165.0494，130.9426 | Chrysin | Flavonoids | TR |
| 79 | 27.45 | C_11_H_20_O | [M+H]+1 | 169.1581 | -0.3 | 113.0594，99.0446，71.0136 | 10-Undecenal | Others | YYR |
| 80 | 27.92 | C_24_H_38_O_4_ | [M-H]-1 | 389.2687 | -1.2 | 277.1447，259.1344，233.1546 | Bis(2-ethylhexyl) phthalate | Saccharides | TR |
| 81 | 28.78 | C_18_H_37_NO_2_ | [M+H]+1 | 300.2887 | -1.5 | 284.2945，267.2682，197.2262 | Sphingosine | Organic acids | LG |
| 82 | 29.90 | C_16_H_22_O_4_ | [M-H]-1 | 277.1439 | -0.3 | 134.0361，121.0286 | Dibutyl phthalate | Saccharides | TR |
| 83 | 30.14 | C_18_H_30_O_2_ | [M+H]+1 | 279.2309 | -3.4 | 261.2203，123.1167 | α-Linolenic acid | Saccharides | TR |

**Table. S2 Characterization of chemical compounds in QJWJTG (continued)**

| **NO.** | **RT (min)** | **Formula** | **Adduct** | **m/z** | **Error (ppm)** | **MS/MS** | **Compounds** | **Structure type** | **Source** |
| --- | --- | --- | --- | --- | --- | --- | --- | --- | --- |
| 84 | 30.88 | C_21_H_40_O_4_ | [M+H]+1 | 357.2983 | -3.0 | 247.2413，135.1164，109.1011 | Monoolein | Flavonoids | DGZ |
| 85 | 31.13 | C_18_H_34_O_3_ | [M+H]+1 | 299.2574 | -2.2 | 279.2327，183.1383 | 12-Hydroxy-9-octadecenoic acid | Saccharides | DGZ，YYR |
| 86 | 31.90 | C_16_H_30_O_2_ | [M+H]+1 | 255.2311 | -3.0 | 142.3848，67.0838 | Palmitoleic acid | Saccharides | YYR |
| 87 | 32.51 | C_18_H_32_O_3_ | [M-H]-1 | 295.2273 | -0.1 | 277.2164，195.1376，171.1013 | 13-HODE | Saccharides | TR |
| 88 | 33.58 | C_18_H_32_O_2_ | [M-H]-1 | 279.2320 | -1.5 | 261.2215，239.4934 | Linoleic acid | Phenolic Acids | YYR |
| 89 | 33.90 | C_18_H_36_O_2_ | [M+H]+1 | 285.2774 | -3.0 | 257.2681，239.2364，221.2266， | Ethyl palmitate | Phenolic Acids | LG |
| 90 | 35.01 | C_14_H_28_O_2_ | [M-H]-1 | 227.2006 | -2.2 | 99.7051，75.4018 | Myristic acid | Alkaloids | LG |
| 91 | 37.32 | C_30_H_48_O_3_ | [M-H]-1 | 455.3520 | -1.1 | 305.2483，237.2378，261.2582 | Oleanolic acid | Phenolic Acids | YYR |
| 92 | 37.82 | C_20_H_38_O_2_ | [M+H]+1 | 311.2936 | -2.8 | 265.2523，237.2572，223.2429 | Ethyl oleate | Phenolic Acids | TR，YYR，LG |
| 93 | 43.92 | C_15_H_30_O_2_ | [M-H]-1 | 241.2165 | -1.1 | 223.2606，197.2274，59.0137 | Pentadecanoic acid | Others | LG，YYR |
| 94 | 44.16 | C_16_H_32_O_2_ | [M-H]-1 | 255.2320 | -1.6 | 255.2323，120.3208 | Hexadecanoic acid | Flavonoids | DGZ，LG |
| 95 | 47.03 | C_20_H_34_O_2_ | [M-H]-1 | 305.2477 | -1.2 | 287.2389，261.2588 | Eicosatrienoic acid | Phenolic Acids | LG，TR，YYR |

**Table. S2 Characterization of chemical compounds in QJWJTG (continued)**

| NO. | RT (min) | Formula | Adduct | m/z | Error (ppm) | MS/MS | Compounds | Structure type | Source |
| --- | --- | --- | --- | --- | --- | --- | --- | --- | --- |
| 96 | 53.19 | C_22_H_42_O_4_ | [M+H]+1 | 371.3145 | -2.9 | 129.0547，111.0448，101.0598 | Bis(2-ethylhexyl)adipate | Phenolic Acids | TR，YYR |
| 97 | 54.37 | C_19_H_38_O_2_ | [M-H]-1 | 297.2791 | -0.9 | 253.2536，183.0117 | Nonadecylic acid | Terpenoids and Steroids | TR，YYR，LG，DGZ |

**Table. S3 Absorbed components of QJWJTD in mouse plasma**

| **NO.** | **RT (min)** | **Formula** | **Adduct** | **m/z** | **Error (ppm)** | **MS/MS** | **Compounds** | **Structure type** | **Source** |
| --- | --- | --- | --- | --- | --- | --- | --- | --- | --- |
| 1 | 1.10 | C_6_H_13_N_3_O_3_ | [M+H]+1 | 176.1032 | 4.4 | 161.0325，143.0030，72.6792 | Citrulline | Amino Acids and Derivatives | DGZ |
| 2 | 1.16 | C_5_H_11_NO_2_ | [M+H]+1 | 118.0868 | 4.6 | 105.0466，95.0497，70.0393 | Betaine | Alkaloids | TR |
| 3 | 1.21 | C_7_H_7_NO_2_ | [M+H]+1 | 138.0552 | 1.8 | 120.0445，94.0401 | Trigonelline | Alkaloids | DGZ |
| 4 | 1.40 | C_6_H_8_O_7_ | [M-H]-1 | 191.0184 | -4.1 | 129.0181，111.0076，87.0075，85.0282 | Citric acid | Organic acids | YYR |
| 5 | 1.66 | C_7_H_6_O_4_ | [M-H]-1 | 153.0187 | -0.5 | 109.0283，81.0333 | Protocatechuic acid | Phenolic Acids | YYR |
| 6 | 2.00 | C_7_H_6_O_5_ | [M-H]-1 | 169.0137 | 0.6 | 125.0236，97.0286，81.0329 | Gallic acid | Phenolic Acids | YYR |
| 7 | 2.05 | C_7_H_12_O_6_ | [M-H]-1 | 191.0552 | -1.9 | 173.0445，127.0379 | Quinic acid | Organic acids | YYR |
| 8 | 2.85 | C_8_H_7_NO_3_ | [M+H]+1 | 166.0496 | -1.6 | 148.0399，138.0556，123.0443 | Coixol | Others | LG |
| 9 | 3.36 | C_16_H_18_O_9_ | [M-H]-1 | 353.0866 | -1.9 | 191.0551，179.0348，135.0439 | Chlorogenic acid | Phenolic Acids | TR，YYR |
| 10 | 6.51 | C_10_H_13_N_5_O_4_ | [M+H]+1 | 268.1048 | 2.9 | 136.0615，85.0283 | Adenosine | Alkaloids | YYR |
| 11 | 8.06 | C_6_H_11_NO | [M+H]+1 | 114.0916 | 2.3 | 96.0809，85.0652，69.0704 | Caprolactam | Others | YYR |
| 12 | 8.33 | C_20_H_27_NO_11_ | [M+H]+1 | 458.1649 | -0.5 | 147.0490，87.0306 | Amygdalin | Saccharides | TR |
| 13 | 9.06 | C_10_H_10_O_4_ | [M-H]-1 | 193.0497 | -2.0 | 178.0494，149.0593，134.0360 | Ferulic acid | Phenolic Acids | YYR，TR，LG |
| 14 | 9.66 | C_9_H_6_O_4_ | [M+H]+1 | 179.0330 | -4.9 | 164.9836，147.0431 | Esculetin | Others | TR，YYR |

**Table. S3 Absorbed components of QJWJTD in mouse plasma (continued)**

| **NO.** | **RT (min)** | **Formula** | **Adduct** | **m/z** | **Error (ppm)** | **MS/MS** | **Compounds** | **Structure type** | **Source** |
| --- | --- | --- | --- | --- | --- | --- | --- | --- | --- |
| 15 | 10.41 | C_9_H_8_O_3_ | [M+H]+1 | 165.0541 | 0.0 | 148.0285，138.0492，121.0491 | p-Coumaric acid | Phenolic Acids | TR，YYR，TR，LG |
| 16 | 10.94 | C_14_H_17_NO_6_ | [M+H]+1 | 296.1128 | -0.2 | 132.0453，105.348，77.0394 | Prunasin | Saccharides | TR |
| 17 | 11.50 | C_15_H_10_O_7_ | [M+H]+1 | 303.0484 | -3.2 | 180.9996，153.0044 | Quercetin | Flavonoids | DGZ，YYR，TR |
| 18 | 16.35 | C_7_H_6_O_2_ | [M+H]+1 | 123.0443 | 2.0 | 95.0269，80.3847 | Benzoic acid | Organic acids | TR |
| 19 | 16.67 | C_28_H_34_O_15_ | [M-H]-1 | 609.1818 | -0.2 | 489.1402，301.0717，258.0584 | hesperidin | Saccharides | YYR |
| 20 | 17.99 | C_15_H_12_O_6_ | [M-H]-1 | 287.0554 | -0.6 | 243.0653，174.0914 | Eriodictyol | Flavonoids | TR，YYR |
| 21 | 19.52 | C_15_H_14_O_5_ | [M+H]+1 | 275.0904 | -3.6 | 275.0903，151.0386 | Phloretin | Flavonoids | YYR |
| 22 | 20.18 | C_16_H_12_O_7_ | [M-H]-1 | 315.0503 | -0.6 | 300.0276，253.0895，209.1912 | Isorhamnetin | Flavonoids | TR |
| 23 | 27.92 | C_24_H_38_O_4_ | [M+H]+1 | 391.2847 | 2.5 | 281.2318，249.1685 | Dioctyl phthalate | Esters | YYR |
| 24 | 28.78 | C_18_H_37_NO_2_ | [M+H]+1 | 300.2888 | -1.2 | 284.2942，267.2682，197.2262 | Sphingosine | Others | DGZ |
| 25 | 30.63 | C_20_H_30_O_2_ | [M+H]+1 | 303.2322 | 2.9 | 257.2267，115.0745 | Eicosapentaenoic acid | Fatty Acids | YYR |
| 26 | 30.99 | C_29_H_50_O | [M+H]+1 | 415.3915 | -4.8 | 397.3827，215.2682，297.2577 | beita-Sitosterol | Terpenoids and Steroids | YYR |
| 27 | 33.07 | C_30_H_48_O_3_ | [M+H]+1 | 457.3688 | 3.8 | 308.2480，240.2378，263.2582 | Oleanolic acid | Organic acids | YYR |

**Table. S3 Absorbed components of QJWJTD in mouse plasma (continued)**

| **NO.** | **RT (min)** | **Formula** | **Adduct** | **m/z** | **Error (ppm)** | **MS/MS** | **Compounds** | **Structure type** | **Source** |
| --- | --- | --- | --- | --- | --- | --- | --- | --- | --- |
| 28 | 33.58 | C_18_H_32_O_2_ | [M+H]+1 | 281.2470 | 0.1 | 261.2219，239.4934 | Linoleic acid | Fatty Acids | YYR，TR，DGZ |
| 29 | 33.90 | C_18_H_36_O_2_ | [M+H]+1 | 285.2774 | -3.0 | 257.2680，221.2266，239.2368 | Ethyl palmitate | Esters | YYR |
| 30 | 34.06 | C_16_H_32_O_2_ | [M-H]-1 | 255.2325 | 0.4 | 255.2320，120.3207 | Hexadecanoic acid | Fatty Acids | TR，YYR，TR，LG |
| 31 | 34.27 | C_22_H_42_O_4_ | [M+H]+1 | 371.3157 | 0.3 | 129.0546，111.0440，101.0598 | Bis(2-ethylhexyl)adipate | Esters | YYR |
| 32 | 34.69 | C_19_H_38_O_2_ | [M-H]-1 | 297.2799 | 1.8 | 253.2536，183.0118 | Nonadecylic acid | Fatty Acids | YYR |
| 33 | 34.91 | C_29_H_48_O | [M+H]+1 | 413.3792 | 3.4 | 177.1631，97.1013，83.0858，57.0705 | Stigmasterol | Terpenoids and Steroids | YYR，LG，DGZ |

**Table. S4 Absorbed components of QJWJTG in mouse plasma**

| **NO.** | **RT (min)** | **Formula** | **Adduct** | **m/z** | **Error (ppm)** | **MS/MS** | **Compounds** | **Structure type** | **Source** |
| --- | --- | --- | --- | --- | --- | --- | --- | --- | --- |
| 1 | 1.03 | C_6_H_14_N_2_O_2_ | [M+H]+1 | 147.1128 | 3.7 | 145.0979，65.2017 | Lysine | Amino Acids and Derivatives | YYR |
| 2 | 1.10 | C_6_H_13_N_3_O_3_ | [M+H]+1 | 176.1024 | -0.1 | 161.0404，143.0101，72.6792 | Citrulline | Amino Acids and Derivatives | DGZ |
| 3 | 1.12 | C_5_H_10_O_5_ | [M+H]+1 | 151.0591 | -3.0 | 91.0331，77.0175，61.0325 | Xylose | Saccharides | YYR，LG |
| 4 | 1.15 | C_6_H_14_O_6_ | [M+H]+1 | 183.0857 | -0.3 | 103.0431，19.0336，73.0325，61.0325 | Mannitol | Saccharides | DGZ |
| 5 | 1.16 | C_5_H_11_NO_2_ | [M+H]+1 | 118.0862 | -0.4 | 105.0457，95.0490 | Betaine | Alkaloids | LG |
| 6 | 1.21 | C_7_H_7_NO_2_ | [M+H]+1 | 138.0552 | 1.8 | 120.0445，94.0401 | Trigonelline | Alkaloids | DGZ |
| 7 | 1.99 | C_7_H_6_O_5_ | [M-H]-1 | 169.0138 | 0.6 | 125.0236，97.0286，81.0329 | Gallic acid | Phenolic Acids | YYR |
| 8 | 2.10 | C_7_H_12_O_6_ | [M-H]-1 | 191.0550 | -2.9 | 173.0444，127.0386，85.0282 | Quinic acid | Organic acids | TR |
| 9 | 2.86 | C_8_H_7_NO_3_ | [M+H]+1 | 166.0491 | -4.6 | 148.0399，138.0551，123.0443 | Coixol | Others | LG，YYR |
| 10 | 3.36 | C_16_H_18_O_9_ | [M-H]-1 | 353.0869 | -1.0 | 191.0552，179.0340，135.0439 | Chlorogenic acid | Phenolic Acids | TR，YYR |
| 11 | 7.81 | C_7_H_6_O | [M+H]+1 | 107.0491 | -0.7 | 106.0651，79.0545 | Benzaldehyde | Aldehydes | TR |
| 12 | 8.06 | C_6_H_11_NO | [M+H]+1 | 114.0916 | 2.3 | 96.0813，85.0656，69.0704 | Caprolactam | Others | YYR |
| 13 | 8.33 | C_20_H_27_NO_11_ | [M+H]+1 | 458.1671 | 4.2 | 147.0595，87.0384 | Amygdalin | Saccharides | TR |
| 14 | 9.06 | C_10_H_10_O_4_ | [M+H]+1 | 195.0641 | -2.8 | 180.0596，151.06985，136.0460 | Ferulic acid | Phenolic Acids | YYR，TR，LG |

**Table. S4 Absorbed components of QJWJTG in mouse plasma (continued)**

| **NO.** | **RT (min)** | **Formula** | **Adduct** | **m/z** | **Error (ppm)** | **MS/MS** | **Compounds** | **Structure type** | **Source** |
| --- | --- | --- | --- | --- | --- | --- | --- | --- | --- |
| 15 | 9.66 | C_9_H_6_O_4_ | [M+H]+1 | 179.0346 | 4.0 | 164.9837，147.0436 | Esculetin | Others | TR，YYR |
| 16 | 10.41 | C_9_H_8_O_3_ | [M+H]+1 | 165.0543 | 1.2 | 147.0388，137.0592，121.0591 | p-Coumaric acid | Phenolic Acids | TR，YYR，LG |
| 17 | 10.64 | C_9_H_10_O_3_ | [M+H]+1 | 167.0695 | -1.3 | 151.0324，139.0710 | Acetovanillone | Others | LG |
| 18 | 11.50 | C_15_H_10_O_7_ | [M-H]-1 | 301.0346 | -0.8 | 178.9977，151.0024 | Quercetin | Flavonoids | DGZ，YYR，TR |
| 19 | 15.34 | C_15_H_20_O_4_ | [M-H]-1 | 263.1283 | -0.1 | 209.1164，191.1058 | Abscisic acid | Terpenoids and Steroids | TR |
| 20 | 16.67 | C_28_H_34_O_15_ | [M-H]-1 | 609.1814 | -0.9 | 489.1407，301.0719，258.0584 | hesperidin | Saccharides | YYR |
| 21 | 19.52 | C_15_H_14_O_5_ | [M+H]+1 | 275.0908 | -2.2 | 275.0908，169.0629，151.0386 | Phloretin | Flavonoids | YYR |
| 22 | 19.80 | C_15_H_12_O_5_ | [M-H]-1 | 271.0607 | 0.2 | 177.0181，151.0024 | Naringenin | Flavonoids | TR |
| 23 | 20.18 | C_16_H_12_O_7_ | [M-H]-1 | 315.0502 | -0.9 | 300.0267，271.1915 | Isorhamnetin | Flavonoids | DGZ |
| 24 | 23.81 | C_15_H_12_O_4_ | [M-H]-1 | 255.0656 | -0.5 | 125.0959，101.0598，67.4514 | Isoliquiritigenin | Flavonoids | YYR |
| 25 | 27.92 | C_24_H_38_O_4_ | [M+H]+1 | 391.2847 | 2.5 | 281.2514，249.1885 | Dioctyl phthalate | Esters | YYR |
| 26 | 30.98 | C_29_H_50_O | [M+H]+1 | 415.3937 | 0.6 | 397.3829，215.2686，297.2577 | beita-Sitosterol | Terpenoids and Steroids | TR，YYR，LG，DGZ |
| 27 | 31.13 | C_18_H_34_O_3_ | [M+H]+1 | 299.2574 | -2.2 | 279.2324，183.1380 | 12-Hydroxy-9-octadecenoic acid | Fatty Acids | DGZ |
| 28 | 31.48 | C_18_H_34_O_2_ | [M+H]+1 | 283.2626 | -2.0 | 129.1523，98.0078 | Elaidic Acid | Fatty Acids | TR，YYR |

**Table. S4 Absorbed components of QJWJTG in mouse plasma (continued)**

| **NO.** | **RT (min)** | **Formula** | **Adduct** | **m/z** | **Error (ppm)** | **MS/MS** | **Compounds** | **Structure type** | **Source** |
| --- | --- | --- | --- | --- | --- | --- | --- | --- | --- |
| 29 | 33.07 | C_30_H_48_O_3_ | [M+H]+1 | 457.3670 | -0.2 | 307.2686，239.2475，263.2786 | Oleanolic acid | Organic acids | LG |
| 30 | 33.32 | C_15_H_30_O_2_ | [M+H]+1 | 243.2315 | 0.8 | 225.2502，199.2873，61.0735 | Pentadecanoic acid | Fatty Acids | YYR，DGZ |
| 31 | 33.91 | C_20_H_34_O_2_ | [M+H]+1 | 307.2627 | 0.3 | 289.281，263.3189，167.2719 | Eicosatrienoic acid | Fatty Acids | YYR |
| 32 | 34.27 | C_22_H_42_O_4_ | [M+H]+1 | 371.3145 | -2.9 | 129.0543，111.0442，101.0598 | Bis(2-ethylhexyl)adipate | Esters | YYR |
| 33 | 34.69 | C_19_H_38_O_2_ | [M-H]-1 | 297.2791 | -0.9 | 253.2536，183.0119 | Nonadecylic acid | Fatty Acids | YYR |
| 34 | 34.91 | C_29_H_48_O | [M+H]+1 | 413.3761 | -4.1 | 97.1018，83.0858，57.0705 | Stigmasterol | Terpenoids and Steroids | YYR，LG，DGZ |

**Table. S5 Primer sequences**

| Gene | Forward primer | Reverse primer |
| --- | --- | --- |
| RSV-F | GAGGGGAATACTAATGGGTCATAGAA | TCAAATAAAAGGGG TGGACACTGT |
| Mouse TNF-α | GGGAGCAAAGGTTCAGTGAT | CCTGGCCTCTCTACCTTGTT |
| Mouse IL-6 | CTGACAATATGAATGTTGGG | TCCAAGAAACCATCTGGCTAGG |
| Mouse IL-1β | GAAATGCCACCTTTTGACAGTG | GAAATGCCACCTTTTGACAGTG |
| Mouse IFN-γ | GCCTGATTGTCTTTCAAGACTTCAA | TAACTCAAGTGGCATAGATGTGGAA |
| Mouse GAPDH | GTCATTGAGAGCAATGCCAG | GTGTTCCTACCCCCAATGTG |
